# The transcription factor LafI integrates control of flagellar motility and T3SS1-mediated virulence in *Vibrio parahaemolyticus*

**DOI:** 10.1128/aem.01123-26

**Published:** 2026-07-29

**Authors:** Xiaojun Zhong, Manlin Zhang, Jinjie Ye, Ranran Lu, Yuanyang Xu, Zeyu Ma, Junyang Zhao, Wenting Wu, Yameng Bai, Zhexiang Li, Yue Zhang, Li Liu, Xiujuan Zhou, Huochun Yao, Guangzhi Xu, Fenxia Fan, Yinchu Zhu, Menghua Yang

**Affiliations:** 1 College of Animal Science and Technology, College of Veterinary Medicine, Zhejiang A & F University 722545, Hangzhou, China; 2 Key Laboratory of Applied Technology on Green-Eco-Healthy Animal Husbandry of Zhejiang Province; Zhejiang Provincial Engineering Laboratory for Animal Health Inspection & Internet Technology, Zhejiang A & F University 12627, Hangzhou, China; 3 MOE Joint International Research Laboratory of Animal Health and Food Safety, College of Veterinary Medicine, Nanjing Agricultural University 261674, Nanjing, China; 4 State Key Laboratory for Quality and Safety of Agro-Products, Institute of Animal Husbandry and Veterinary Science, Zhejiang Academy of Agricultural Sciences 823338, Hangzhou, China; 5 College of Food and Health, Zhejiang A & F University 726604, Hangzhou, China; 6 State Key Laboratory of Infectious Disease Prevention and Control, National Institute for Communicable Disease Control and Prevention, Chinese Center for Disease Control and Prevention 12415, Beijing, China; Indiana University Bloomington, Bloomington, Indiana, USA

**Keywords:** virulence, motility, type III secretion system, *Vibrio parahaemolyticus*

## Abstract

*Vibrio parahaemolyticus,* a major marine pathogen, employs sophisticated regulatory networks to coordinate motility and virulence during infection, yet the mechanisms remain poorly understood. Here, we identified LafI, a previously uncharacterized transcriptional regulator that represses both motility and virulence in *V. parahaemolyticus* by governing a regulatory circuit between the lateral flagellar system and type III secretion system 1 (T3SS1). We demonstrated that LafI negatively regulates the motility of *V. parahaemolyticus* by inhibiting the expression of both the polar and lateral flagellar systems, while it represses T3SS1-mediated cytotoxicity via the master T3SS1 activator ExsA. Furthermore, we revealed a regulatory circuit wherein LafI represses the lateral flagellar master regulator LafK^L^, which in turn modulates T3SS1 activity through the master activator ExsA, with the latter providing feedback to fine-tune the lateral flagellar system, thereby coordinating *V. parahaemolyticus* virulence and motility. Our work established LafI as a central regulator that couples motility and virulence, offering new insights into bacterial pathogenesis.

**IMPORTANCE:**

Pathogens like *Vibrio parahaemolyticus*, a common cause of seafood-related gastroenteritis, use flagella to move and spread. Although the motilities help bacteria infect hosts, some *V. parahaemolyticus* clinical isolates display swarming deficiency *in vitro* while retaining pathogenicity *in vivo*. The mechanisms by which *V. parahaemolyticus* strains regulate motility and virulence remain unclear. Here, we identified LafI, a previously uncharacterized transcriptional regulator that represses motility and cytotoxicity through a regulatory circuit between the flagellar systems and T3SS1. Our work shows how *V. parahaemolyticus* coordinates motility and virulence to adapt to different environments and provides new insight into the interplay between these critical bacterial processes.

## INTRODUCTION

*Vibrio parahaemolyticus*, a Gram-negative marine pathogen, is a leading cause of seafood-borne acute gastroenteritis, with rising incidence linked to climate change and coastal urbanization (1, 2). The ecological success of this facultative pathogen is due to its remarkable adaptive plasticity. It can exist in marine ecosystems in both planktonic and biofilm states, yet it rapidly transforms into an acute pathogen upon host entry (1). This ability is mediated by sophisticated environmental sensing and gene regulation mechanisms (1, 3, 4). Genes regulating adaptation to the physical and chemical environment are not only key fitness determinants but also form the basis of the virulence arsenal in pathogenic strains (5, 6). Many pathogenic serotypes of *V. parahaemolyticus* have been identified, and they were found to deploy an arsenal of virulence modules, such as the two functionally distinct type III secretion systems (T3SS1 and T3SS2), which mediate cytotoxicity and enterotoxicity, respectively (4, 7–9). While these virulence modules have been well-characterized in *V. parahaemolyticus*, the regulatory mechanism that synchronizes their expression across different growth conditions and diverse strains remains largely unknown.

Within the host, flagellum-mediated motility is a key virulence trait for many pathogens, often requiring functional specialization to navigate diverse environments (10, 11). *V. parahaemolyticus* strains commonly produce two types of flagella: the single polar flagellum for swimming in liquid environments and the numerous lateral flagella for swarming across viscous and surface environments (10). Although *V. parahaemolyticus* upregulates its virulence program during the transition from swimming to swarming motility, a behavioral switch triggered by surface contact that enhances host tissue colonization and damage (12), we found that clinical isolates frequently display phenotypic heterogeneity in swarming motility *in vitro*. The genetic and regulatory underpinnings of this phenotype remain unknown. While bacterial motility is often linked to virulence, it exhibits context-dependent roles across different pathogens. For instance, *Vibrio cholerae* represses flagellar synthesis during host colonization to upregulate toxin production (13, 14), whereas *Pseudomonas aeruginosa* employs motility to disseminate biofilms in chronic infections (15–18). Therefore, the phenotypic heterogeneity observed in clinical isolates suggests that swarming-deficient *V. parahaemolyticus* strains may employ unidentified regulatory mechanisms to compensate for this defect and maintain virulence during host adaptation.

Transcription factors, essential regulators of gene expression found in all living organisms, are widely distributed in bacteria, where they govern diverse processes such as growth, motility, stress tolerance, and virulence (19–21). Here, we report a previously uncharacterized transcription factor, LafI, harboring an HTH_LUXR (SM00421) domain as an important regulator of *V. parahaemolyticus*. Using a clinical *V. parahaemolyticus* strain with a swarming defect, we demonstrated that LafI represses motility and cytotoxicity via a regulatory circuit between the flagella and T3SS1, directly linking motility control to virulence regulation. Our findings improve the understanding of motility-virulence relationships in *Vibrio* species and offer new insights into bacterial pathogenesis.

## RESULTS

### *V*. *parahaemolyticus* clinical strain HZ shows a swarming defect on the surface

**Fig 1:**
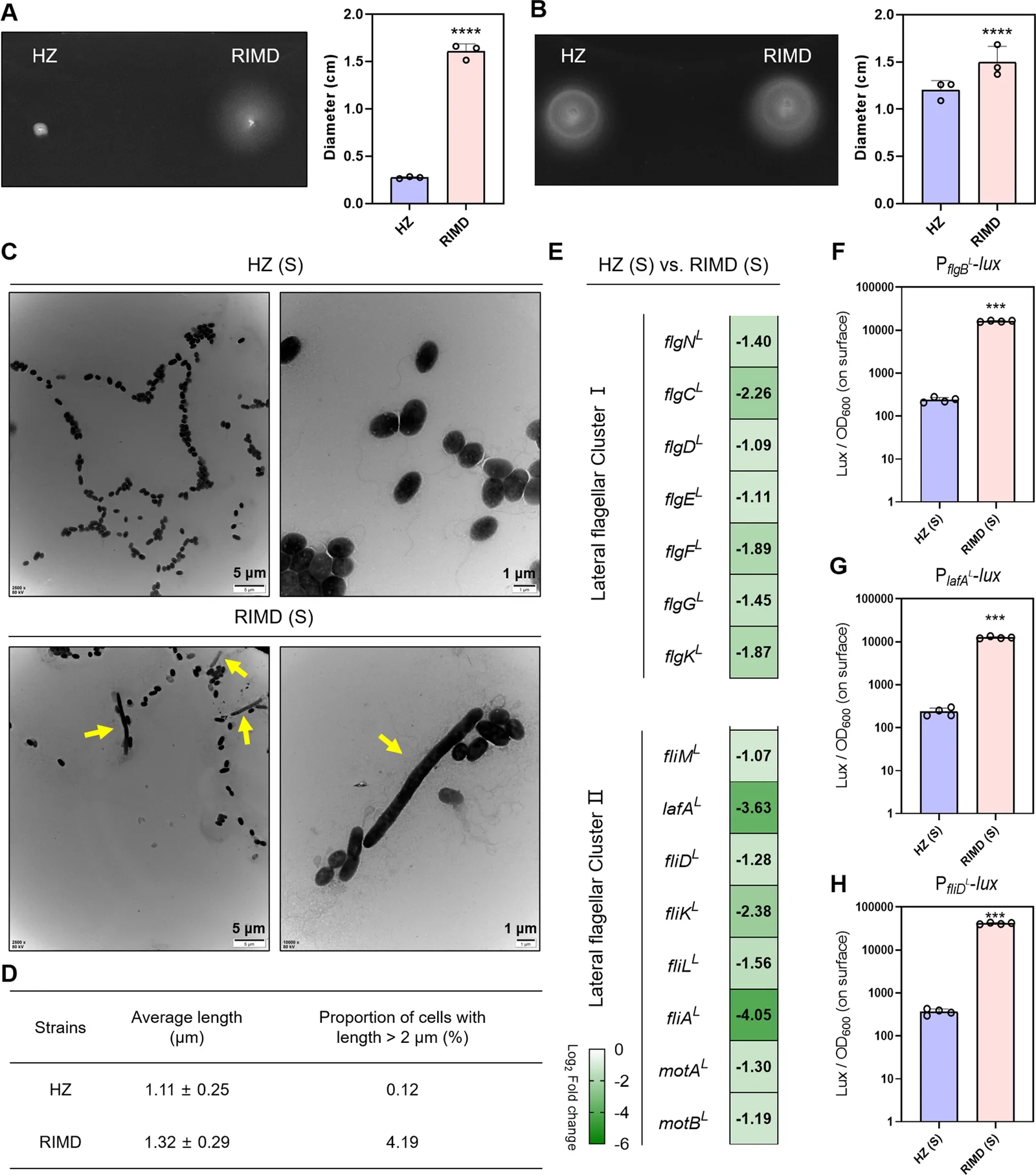
*V. parahaemolyticus* clinical strain HZ shows a swarming defect compared with strain RIMD. (**A**) The swarming colony and the migration diameter of the HZ and RIMD strains. Swarming assays were performed on plates containing 2.5% (wt/vol) BHI broth, 1.2% (wt/vol) agar, and 1.5% (wt/vol) NaCl. (**B**) The swimming colony and the migration diameter of the HZ and RIMD strains. The migration diameter of the swarming colonies was measured by using ImageJ software. The migration of at least three biological replicates of each strain was analyzed. (**C**) Transmission electron micrographs of the HZ and RIMD strains under surface (S) conditions. The yellow arrows show the elongated cell body. The scale bars indicate the magnification size. (**D**) The cell lengths of the HZ and RIMD strains were measured by TEM. At least 100 cells per strain were selected for length quantification. (**E**) Heat map analysis of the relative expression level of the lateral flagellar gene clusters in the HZ and RIMD strains under surface (S) conditions. (**F–H**) The transcription levels of the lateral flagellar gene clusters in the HZ and RIMD strains were assessed by measuring luminescence in the strains containing P*_flgB_^L^-lux*, P*_lafA_^L^-lux*, and P*_fliD_^L^-lux*, respectively. *V. parahaemolyticus* containing promoter-*lux* transcriptional fusion plasmids were grown on an agar surface at 37°C. The unpaired two-tailed Student’s *t*-test was used for statistical analysis (***, *P* < 0.001; ****, *P* < 0.0001).

*V. parahaemolyticus* HZ strain is a clinical isolate from the Zhejiang Provincial Center for Disease Control and Prevention, China (22). By testing the motility of this strain, we found that it could swim in the soft agar yet could not swarm on the agar surface; however, another clinical isolate, RIMD 2210633 (hereafter referred to as RIMD), could both swim and swarm (Fig. 1A and B). Previous studies indicated that surface recognition affects swarmer cell differentiation and induces an elongated cell body (12). Here, elongated cells for the HZ strain were hardly observed by transmission electron microscopy (TEM) compared to the RIMD strain (Fig. 1C), and the average percentage of cells over 2 μm in the HZ strain sample was much lower than that in the RIMD strain sample (Fig. 1D). In addition, the lateral flagella were clearly observed in those elongated cells of the RIMD strain, but could not be observed in the HZ strain (Fig. 1C). These results indicated that the HZ strain fails to synthesize the lateral flagella, which results in a swarming defect. A comparative genomic analysis showed that the HZ strain encodes the complete lateral flagellar gene cluster in its genome, as that of the RIMD strain. However, by comparing the transcriptomics of the HZ strain with the RIMD strain, we found that some of the lateral flagellar genes are significantly down-regulated in the HZ strain under the surface condition (Fig. 1E). By testing the promoter activities of the lateral flagellar gene operons, we further found that the promoters of *flgB^L^*, *lafA^L^*, and *fliD^L^* could not be activated in the HZ strain (Fig. 1F through H). These results indicated that the expression of lateral flagellar genes is inhibited in the HZ strain.

### The transcription factor LafI inhibits *V*. *parahaemolyticus* motility

Previous studies indicated cyclic-di-GMP (c-di-GMP) plays an important role in regulating the swarming motility of *V. parahaemolyticus* by repressing lateral flagellar gene expression (23, 24). To find out whether the swarming defect of the HZ strain is caused by the high level of intracellular c-di-GMP, a plasmid containing the *scrABC* operon, which could downregulate the c-di-GMP levels, was transferred into the HZ and RIMD strains, respectively (25, 26). We found that ScrABC could enhance swarming of the RIMD strain; however, it could not recover the swarming defect of the HZ strain (Fig. S1), which indicated that the swarming defect of the HZ strain is probably not caused by the high level of intracellular c-di-GMP.

**Fig 2:**
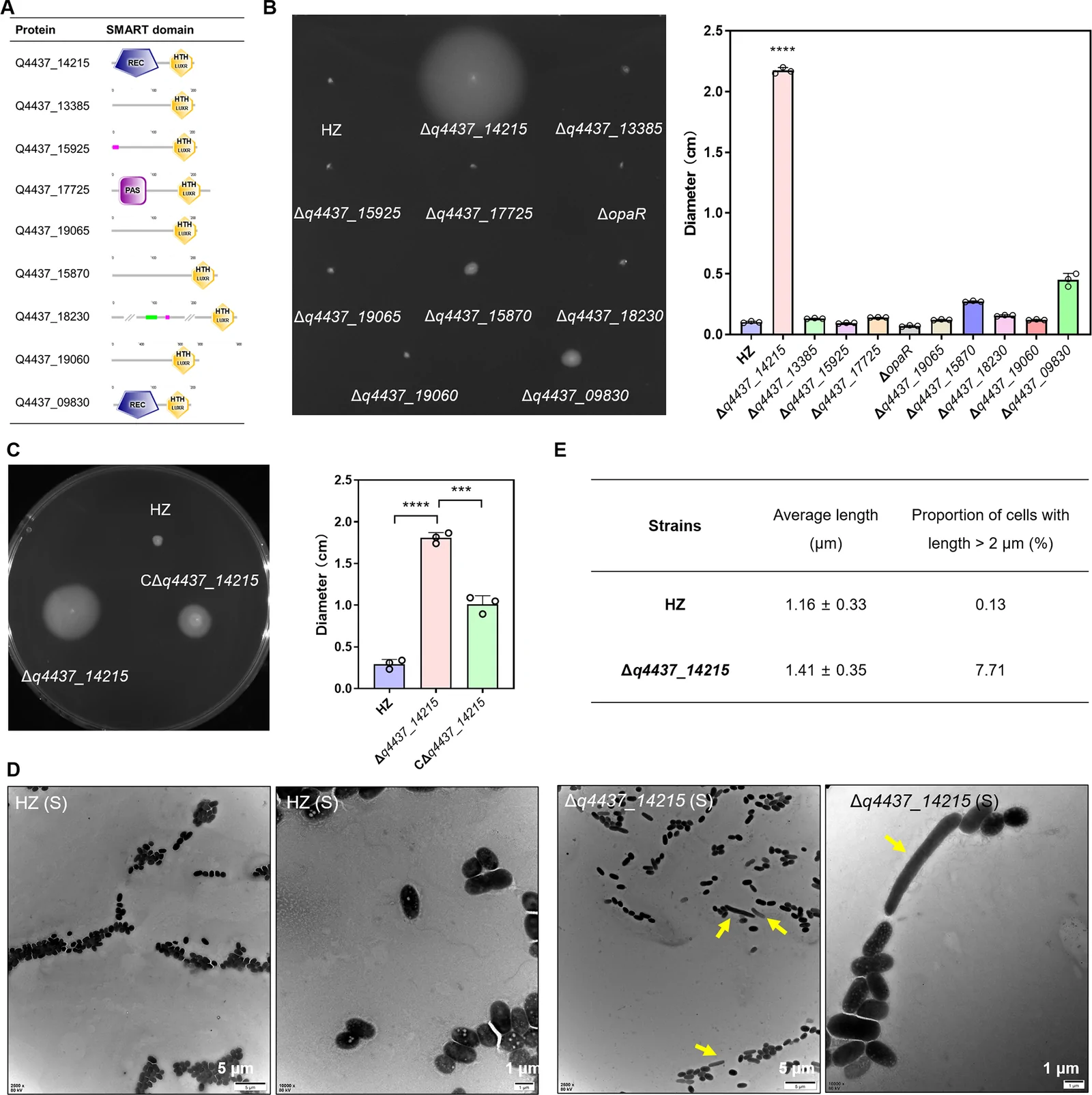
The transcription factor Q4437_14215 inhibits the swarming motility of *V. parahaemolyticus* strain HZ. (**A**) The domain architecture of the nine transcription factors. The domain architectures are based on predictions from the SMART database (https://smart.embl.de/). (**B**) The swarming colony and the migration diameter of the wild-type HZ and the indicated mutant strains. (**C**) The swarming colony and the migration diameter of the wild-type HZ, Δ*q4437_14215*, and CΔ*q4437_14215* strains. The CΔ*q4437_14215* strain carries a complementary plasmid for *q4437_14215*, while the HZ and Δ*q4437_14215* strains harbor an empty pBAD24 plasmid. The migration diameter of the swarming colonies was measured by using ImageJ software. (**D**) Transmission electron micrographs of the wild-type HZ and Δ*q4437_14215* strains under surface conditions. The yellow arrows show the elongated cell body. The scale bars indicate the magnification size. (**E**) The cell lengths of the wild-type HZ and Δ*q4437_14215* strains were measured by TEM. At least 100 cells per strain were selected for length quantification. The unpaired two-tailed Student’s *t*-test was used for statistical analysis (***, *P* < 0.001; ****, *P* < 0.0001).

The inability to activate the promoters of lateral flagellar genes in the *V. parahaemolyticus* HZ strain prompted us to investigate their regulatory mechanisms. Given that transcription factors, particularly those belonging to the LuxR-type family known for coordinating behaviors like motility, often regulate bacterial gene expression (19, 27–30), we focused on this protein class. We identified nine transcription factors harboring a conserved helix-turn-helix LuxR-type (HTH_LUXR, SM00421) DNA-binding domain in the HZ strain genome and constructed gene deletion mutants for each to elucidate their roles (Fig. 2A). We found that the *q4437_14215* gene knock-out mutant (Δ*q4437_14215*) regains the ability of swarming on agar surface, while the swarming motility of the other mutant strains was not obviously different compared with that of the wild-type HZ strain (Fig. 2B). The growth curves displayed no significant difference between the Δ*q4437_14215* mutant and the wild-type HZ strain, and the swarming ability was decreased when a complementary plasmid was introduced into Δ*q4437_14215* (Fig. S2A and C), which confirms the regulation of the swarming motility by Q4437_14215. Unlike the wild-type HZ strain, elongated cells for the Δ*q4437_14215* mutant could be observed in a high percentage by TEM (Fig. 2D), and the average percentage of cells over 2 μm in the Δ*q4437_14215* mutant sample was even higher than that in the RIMD strain sample (Fig. 1D and 2E). Intriguingly, we found that Q4437_14215 had the same effect on swimming motility as it did on swarming motility of the wild-type HZ strain (Fig. S2B), which indicated that Q4437_14215 inhibits the motility of the HZ strain, either when growing in liquid or on a surface. Based on these results, we named Q4437_14215 as LafI (lateral flagella inhibitor) hereafter.

### LafI inhibits motility by regulating flagellar expression

**Fig 3:**
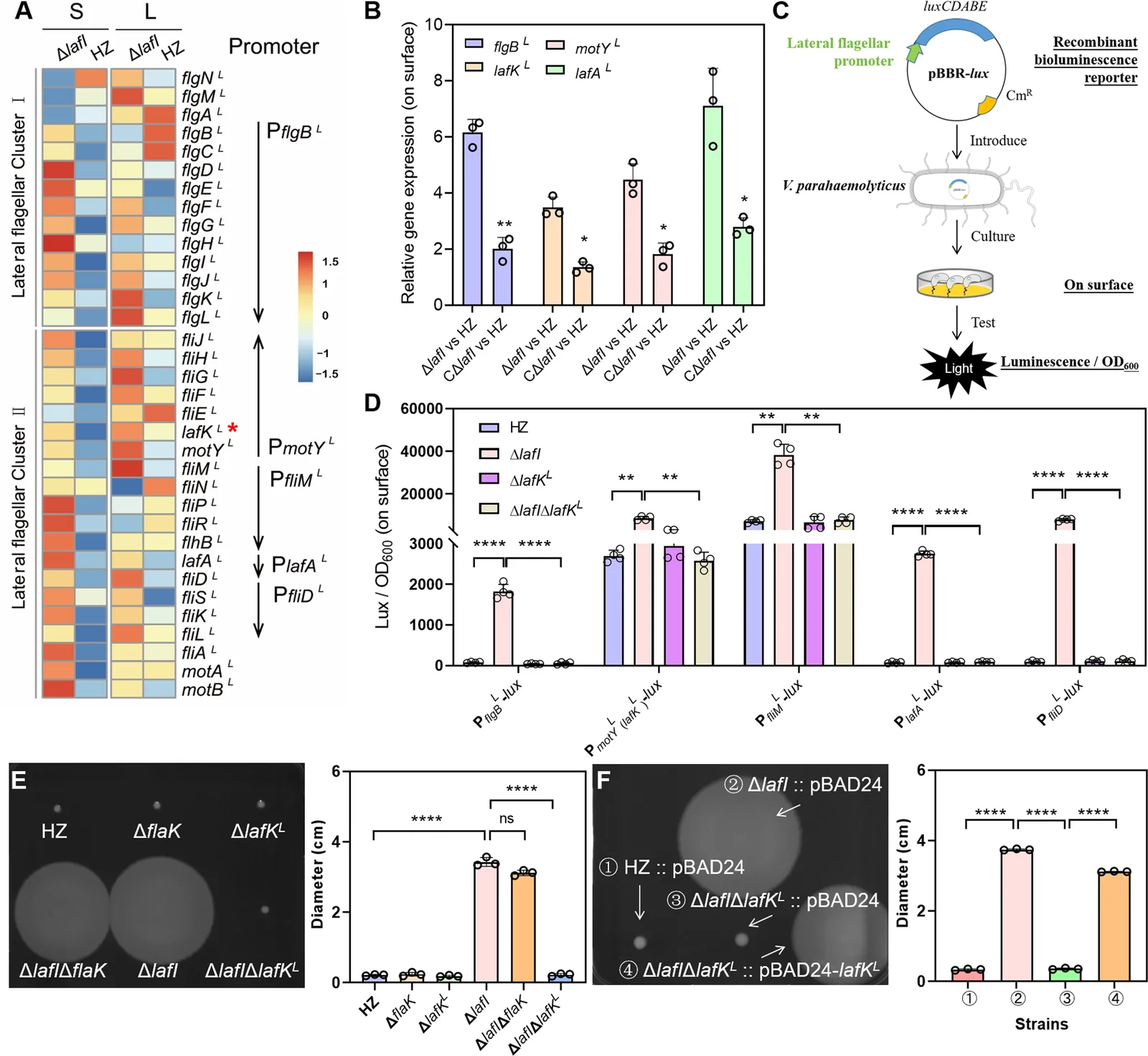
LafI regulates the expression of the lateral flagella. (**A**) Heat map analysis of the relative expression level of the lateral flagellar gene clusters in the Δ*lafI* and wild-type HZ strains under the surface (S) and liquid (L) conditions. The expression values were Z-score normalized to a range of −1.5 to 1.5. The arrows indicate the transcription direction of the operon. (**B**) The mRNA levels of the lateral flagellar genes in the wild-type HZ, Δ*lafI*, and CΔ*lafI* strains were determined by RT-qPCR. The CΔ*lafI* strain carries a complementary plasmid for *lafI*, while the HZ and Δ*lafI* strains harbor an empty pBAD24 plasmid. The results of RT-qPCR are expressed as means ± SD from three independent experiments. (**C**) The workflow for evaluating the promoter activities of the lateral flagellar genes based on a bioluminescence reporter assay. (**D**) The transcription levels of the lateral flagellar gene clusters in the wild-type HZ and indicated mutant strains were assessed by measuring luminescence in the strains containing P*_flgB_^L^-lux*, P*_motY_^L^-lux*, P*_fliM_^L^-lux*, P*_lafA_^L^-lux*, and P*_fliD_^L^-lux*, respectively. (**E and F**) The swarming colony and the migration diameter of the wild-type HZ and the indicated mutant strains. The migration diameter of the swarming colonies was measured by using ImageJ software. The unpaired two-tailed Student’s *t*-test (**B**) and the one-way ANOVA, followed by Tukey’s test (**D–F**), were used for statistical analysis (ns, not significant, *P* > 0.05; *, *P* < 0.05; **, *P* < 0.01; ****, *P* < 0.0001).

To estimate the generality of prokaryotes whose genomes encode *lafI*, we then screened the National Center for Biotechnology Information (NCBI) database for LafI orthologs, which were further subjected to a phylogenetic analysis. As shown in Fig. S3A, although LafI exists widely among the *Proteobacteria*, we found that it is conserved in *Vibrio* species, and its function remains largely unknown. To further investigate the molecular mechanisms by which LafI regulates the motility of *V. parahaemolyticus*, RNA sequencing (RNA-seq) was performed, and the transcriptomes of the wild-type HZ and Δ*lafI* strains were compared during growth in liquid and on the surface (Tables S1 and S2). Transcriptomic analysis of LafI showed that the lateral flagellar genes were significantly up-regulated in the Δ*lafI* strain under both the liquid and surface conditions, whereas the polar flagellar genes were up-regulated exclusively in liquid conditions (Fig. 3A; Fig. S3B and S4A). To confirm that deletion of *lafI* was responsible for the upregulation of the flagellar genes in the mutant, we checked the mRNA levels of the flagellar genes in the complemented strain CΔ*lafI*, which contains a pBAD24 plasmid overproducing LafI. As shown in Fig. 3; Fig. S4B, the expression of the lateral flagellar genes, including *lafK^L^*, and polar flagellar genes was significantly upregulated in the Δ*lafI* strain and partially restored in the CΔ*lafI* strain. Consistently, bioluminescence reporter assays also showed that the promoter activities of lateral flagellar operons, including the *motY^L^-lafK^L^* operon (P*_motY_^L^-lux*), and polar flagellar operons were significantly increased in the Δ*lafI* strain compared with the wild-type HZ strain (Fig. 3C and D; Fig. S4C).

LafK^L^ is a key σ^54^-dependent flagellar regulator required for *V. parahaemolyticus* swarming motility (31). To investigate the mechanism by which LafI regulates expression of the lateral flagella, we knocked out *lafK^L^* in the wild-type or Δ*lafI* genetic background of the HZ strain and measured the activity of the lateral flagellar promoter-*lux* fusions in these strains cultured on the surface (Fig. 3C). We found that LafI could not affect the expression of the lateral flagellar genes when *lafK^L^* was deleted (Fig. 3D). We also knocked out *flaK*, which encodes an important regulator of the polar flagellum in the wild-type or Δ*lafI* genetic background of the HZ strain, and further observed the motility of the appropriate deletion strains. We found that the deletion of *lafK^L^* caused Δ*lafI* to lose the ability to swarm, whereas the deletion of *flaK* did not significantly affect the swarming motility of Δ*lafI* (Fig. 3E). All those mutants containing a deletion of *flaK* lost the ability to swim, whereas the deletion of *lafK^L^* did not affect the swimming motility of the wild-type HZ or Δ*lafI* strains (Fig. S4D). In addition, we found that overexpression of LafK^L^ in the Δ*lafI*Δ*lafK^L^* strain restored swarming motility, while overexpression of FlaK in the Δ*lafI*Δ*lafK^L^* strain restored swimming motility (Fig. 3F; Fig. S4E). These results suggested that LafI represses the swimming motility of *V. parahaemolyticus* by inhibiting the expression of the polar flagellar gene, while it negatively regulates the swarming motility by inhibiting the lateral flagellar gene expression through LafK^L^, and there is no cross-regulation between the two types of motilities mediated by LafI.

### LafI regulates *V*. *parahaemolyticus* virulence by inhibiting T3SS1 expression

**Fig 4:**
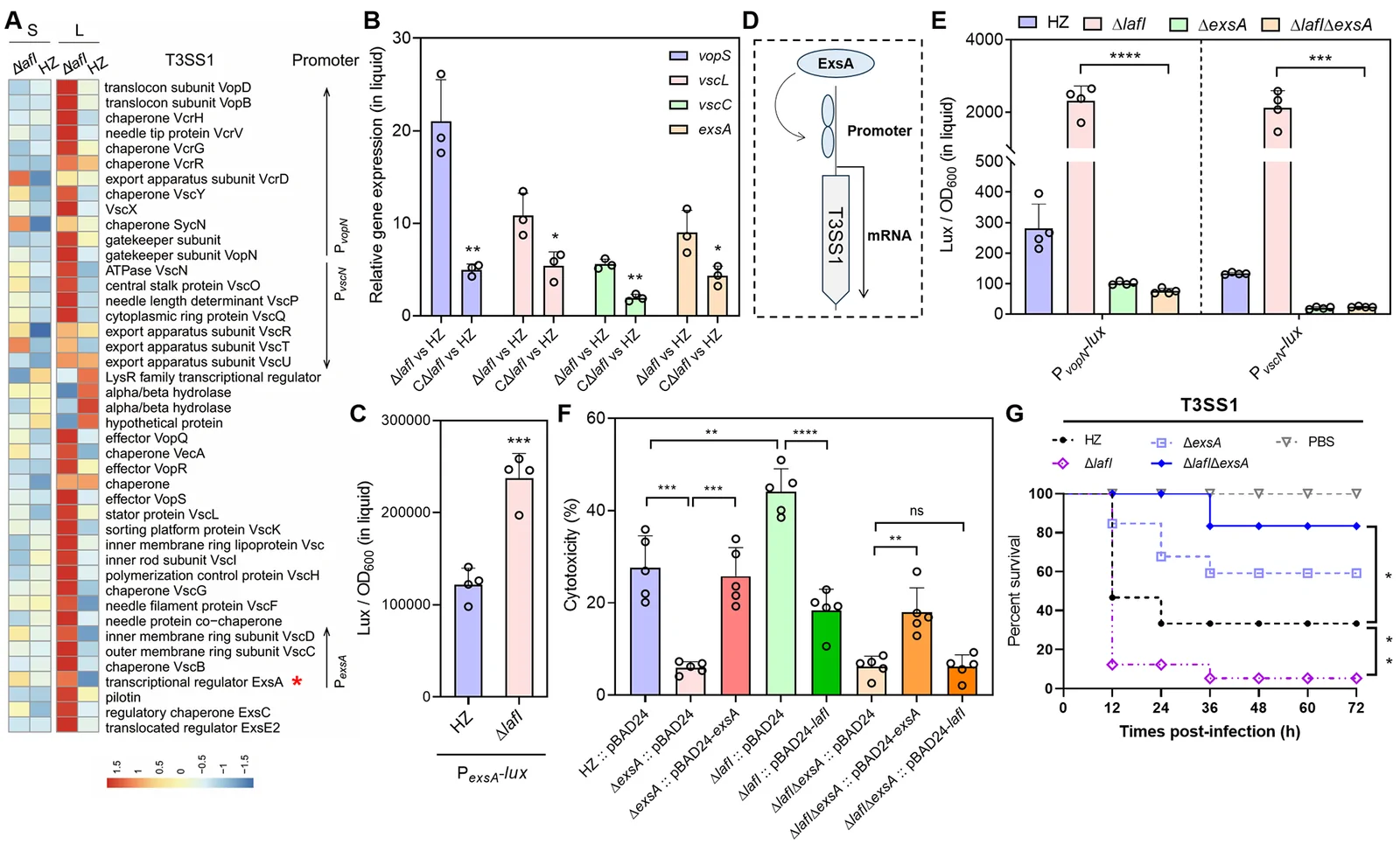
LafI regulates the T3SS1 expression and the virulence of *V. parahaemolyticus*. (**A**) Heat map analysis of the relative expression level of the T3SS1 gene clusters in the Δ*lafI* and wild-type HZ strains under the surface (S) and liquid (L) conditions. The expression values were Z-score normalized to a range of −1.5 to 1.5. The arrows indicate the transcription direction of the operon. (**B**) The mRNA levels of T3SS1 genes in the wild-type HZ, Δ*lafI*, and CΔ*lafI* strains were determined by RT-qPCR. The CΔ*lafI* strain carries a complementary plasmid for *lafI*, while the HZ and Δ*lafI* strains harbor an empty pBAD24 plasmid. The results of RT-qPCR are expressed as means ± SD from three independent experiments. (**C and E**) The transcription levels of the T3SS1 gene clusters in the wild-type HZ and the indicated mutant strains were assessed by measuring luminescence in the strains containing promoter-*lux* transcriptional fusion plasmids. Bacterial cultures were grown in liquid at 37°C. (**D**) ExsA-mediated regulation of T3SS1 gene expression. (**F**) LDH assay results (% cytotoxicity) for HeLa cells infected with the wild-type HZ and the indicated mutant strains. (**G**) Survival curves of zebrafish infected with the wild-type HZ and the indicated mutant strains. Zebrafish cohorts were intraperitoneally inoculated with designated bacterial strains and monitored for mortality over a 72-h period. Control cohorts received an equivalent volume of sterile PBS. The unpaired two-tailed Student’s *t*-test (**B and C**), the one-way ANOVA, followed by Tukey’s test (**E and F**), and the log-rank (Mantel-Cox) test (**G**) were used for statistical analysis (ns, not significant, *P* > 0.05; *, *P* < 0.05; **, *P* < 0.01; ***, *P* < 0.001; ****, *P* < 0.0001).

Further investigation of the regulon of LafI obtained by RNA-seq analyses found that the gene cluster of T3SS1 was significantly up-regulated in Δ*lafI* under the liquid condition (Fig. 4A; Fig. S3B). We randomly selected four genes to perform RT-qPCR for verification; the expression of these genes was consistent with the transcriptomic data and also restored in the complemented strain CΔ*lafI* (Fig. 4B), which confirmed that LafI was responsible for the up-regulation of T3SS1 in the Δ*lafI* strain. Notably, the expression of the T3SS1 master regulator ExsA was also up-regulated in the Δ*lafI* strain (Fig. 4A and B). We constructed the promoter-*lux* fusions P*_exsA_-lux* and found that its promoter activity was significantly increased in the Δ*lafI* strain (Fig. 4C). ExsA controls T3SS1 expression in *V. parahaemolyticus* by regulating promoter activity (Fig. 4D). To further investigate the relationship between LafI and ExsA in regulating T3SS1 expression, we generated the double gene mutant strain of Δ*lafI*Δ*exsA*. By comparing the promoter activity of the promoter-*lux* fusions of the T3SS1 gene cluster in these strains, we found that LafI could not affect the expression of the T3SS1 when *exsA* was deleted (Fig. 4E). These results suggested that LafI inhibits the expression of the T3SS1 genes through ExsA.

As T3SS1 has been proven to contribute to *V. parahaemolyticus*-induced cytotoxicity (32), we then evaluated whether LafI regulates the cytotoxicity by using the HeLa cell model. The lactate dehydrogenase (LDH) release assays showed that the LDH level of the Δ*lafI* strain was significantly higher than that of the wild-type HZ and Δ*lafI*Δ*exsA* strains (Fig. 4F). In addition, complementation of Δ*lafI* with pBAD24-*lafI* and Δ*exsA* with pBAD24-*exsA* restored cytotoxicity to levels comparable to those of the wild-type HZ strain, whereas in the Δ*lafI*Δ*exsA* strain, complementation with pBAD24-*exsA*, but not pBAD24-*lafI*, restored cytotoxicity (Fig. 4F). To investigate whether the up-regulation of T3SS1 in Δ*lafI* would affect the virulence of *V*. *parahaemolyticus*, the zebrafish model was utilized in this study (33). The survival rate of the zebrafish infected by the Δ*lafI* strain was significantly lower than that infected by the wild-type HZ strain (Fig. 4G); however, in the absence of a functional ExsA, LafI no longer inhibited the virulence of *V. parahaemolyticus*, because we observed that the survival rate of the zebrafish infected by the Δ*lafI*Δ*exsA* mutant was even higher than that infected by Δ*exsA* (Fig. 4G). These results indicated that LafI regulates *V. parahaemolyticus* cytotoxicity and virulence by inhibiting the T3SS1 expression.

### LafI regulates T3SS1-mediated virulence through LafK^L^, with ExsA feedback fine-tuning swarming motility

**Fig 5:**
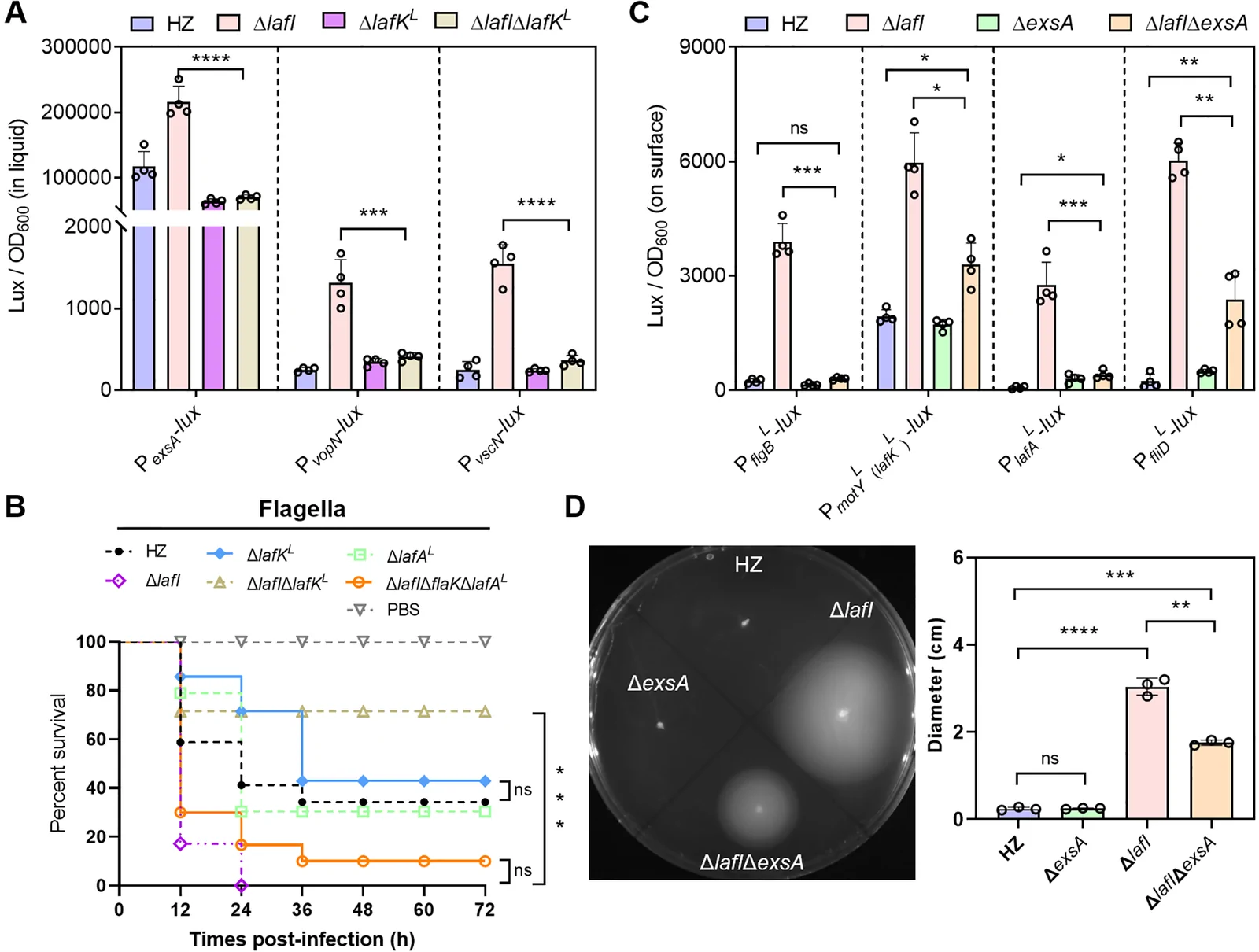
LafI regulates virulence and swarming motility of *V. parahaemolyticus* through LafK^L^ and ExsA. (**A**) The transcription levels of the T3SS1 gene clusters in the wild-type HZ and the indicated mutant strains were assessed by measuring luminescence in the strains containing promoter-*lux* transcriptional fusion plasmids. Bacterial cultures were grown in liquid at 37°C. (**B**) Survival curves of zebrafish infected with the wild-type HZ and the relevant mutants. Zebrafish cohorts (*n* = 15 per group) were intraperitoneally inoculated with designated bacterial strains (2.0 × 10^7^ CFU per fish) and monitored for mortality over a 72-h period. Control cohorts received an equivalent volume of sterile PBS. (**C**) The transcription levels of the lateral flagellar gene clusters in the wild-type HZ and the indicated mutant strains were assessed by measuring luminescence in the strains containing P*_flgB_^L^-lux*, P*_motY_^L^-lux*, P*_lafA_^L^-lux*, and P*_fliD_^L^-lux*, respectively. *V. parahaemolyticus* containing promoter-*lux* transcriptional fusion plasmids were grown on an agar surface at 37°C. (**D**) The swarming colony and the migration diameter of the wild-type HZ and the indicated mutant strains. The migration diameter of the swarming colonies was measured by using ImageJ software. The one-way ANOVA, followed by Tukey’s test (**A, C, and D**) and the log-rank (Mantel-Cox) test (**B**), was used for statistical analysis (ns, *P* > 0.05; *, *P* < 0.05; **, *P* < 0.01; ***, *P* < 0.001; ****, *P* < 0.0001).

A previous study suggested that LafK^L^ appeared to be one regulatory link between swarming and T3SS1 gene expression in *V. parahaemolyticus* (34). Considering LafI was a T3SS1 inhibitor and repressed the *lafK^L^* expression (Fig. 3 and 4), we then investigated the relationship between LafI and LafK^L^ in the regulation of T3SS1 expression by measuring the activity of the promoter-*lux* fusions of the T3SS1 gene cluster in the HZ and appropriate deletion strains cultured in liquid. We found that LafI could not affect the expression of T3SS1 when *lafK^L^* was deleted (Fig. 5A); meanwhile, the survival rate of zebrafish infected with Δ*lafI*Δ*lafK^L^* was significantly higher than that infected by the Δ*lafI* strain (Fig. 5B). To further investigate whether the polar or lateral flagella mediate the effect of LafI on virulence, we knocked out *lafA^L^*, which encodes lateral flagellin in both wild-type and Δ*lafI*Δ*flaK* backgrounds of the HZ strain, followed by virulence assessment. We observed that the survival rates of zebrafish infected with Δ*lafI*Δ*flaK*Δ*lafA^L^* and Δ*lafI* were comparable, and no significant difference was observed between Δ*lafA^L^* and the wild-type HZ strain (Fig. 5B). These results demonstrated that the up-regulated structural flagellar genes in Δ*lafI* did not contribute to the virulence of *V. parahaemolyticus*, whereas the flagellar regulator LafK^L^, when derepressed from LafI inhibition, promoted virulence via T3SS1.

Intriguingly, under surface conditions, bioluminescence reporter assays revealed that the promoter activities of lateral flagellar genes in the Δ*lafI*Δ*exsA* strain were significantly lower than those in the Δ*lafI* strain but higher than those in the wild-type HZ strain (Fig. 5C). By comparing the swarming motility of the wild-type HZ and appropriate deletion strains, we further found that if knocking out *exsA* in the wild-type background, the swarming motility of the mutant was unchanged; however, the swarm ability of the Δ*lafI*Δ*exsA* strain was significantly decreased compared to that of the Δ*lafI* strain but increased compared to that of the wild-type HZ strain (Fig. 5D), which was consistent with the data of bioluminescence reporter assays. Our results suggested that the T3SS1 master regulator ExsA fine-tunes swarming motility in the Δ*lafI* background through its feedback on the lateral flagellar system. This finding reveals a regulatory circuit between these systems and provides a functional extension to the previously reported LafK^L^-ExsA regulatory cascade.

## DISCUSSION

**Fig 6:**
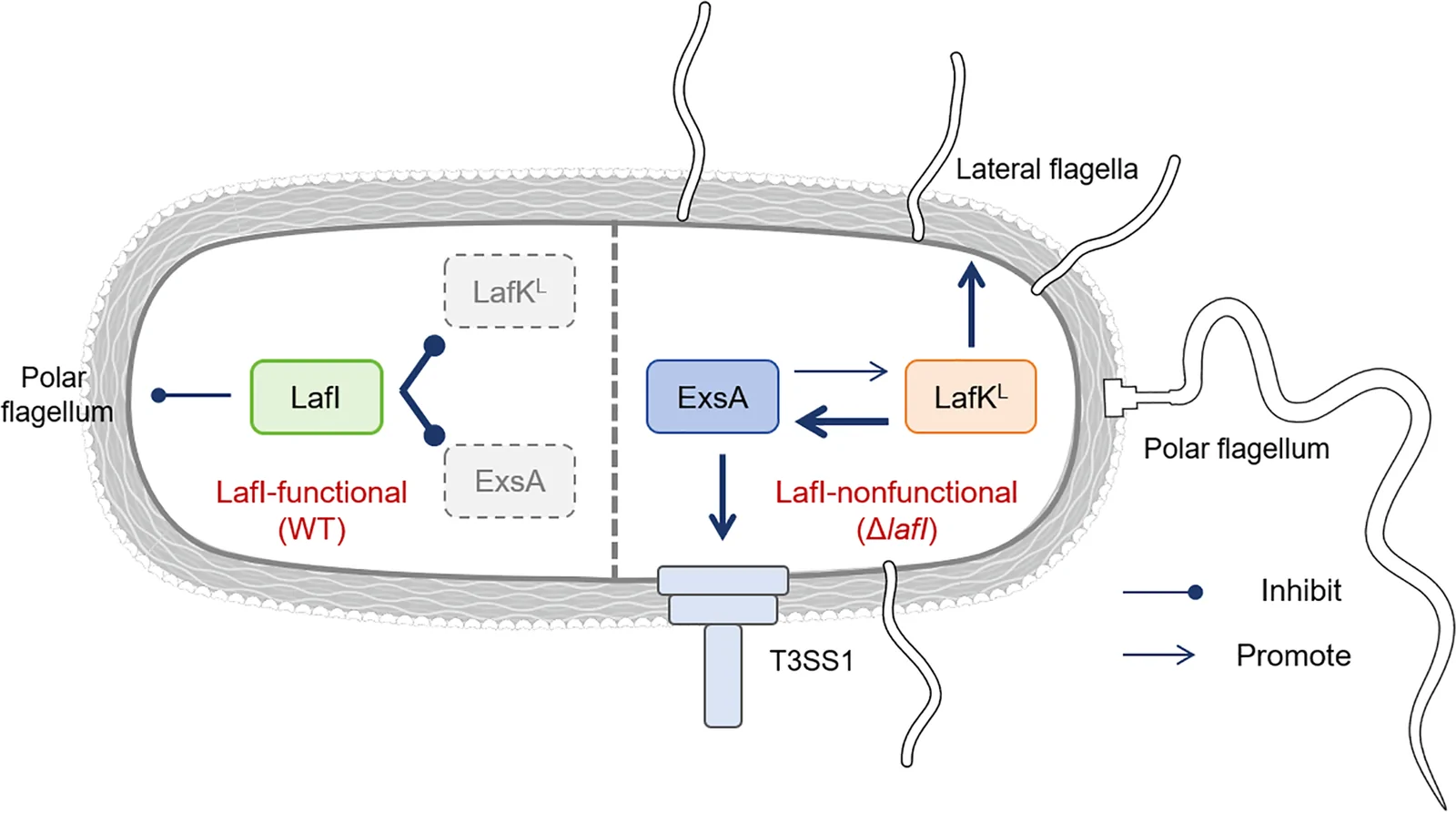
A regulatory model for LafI-mediated coordination of *V. parahaemolyticus* motility and virulence. LafI is proposed to be activated by unknown signals to repress the expression of both flagellar systems and T3SS1. In the *V. parahaemolyticus* Δ*lafI* mutant, the derepression of the flagellar regulator LafK^L^ leads to the activation of lateral flagella (enabling swarming) and upregulation of the T3SS1 master regulator ExsA, which in turn enhances bacterial virulence by activating T3SS1 gene expression. Furthermore, ExsA provides feedback to fine-tune the lateral flagellar system to regulate the bacterial swarming motility on surfaces. Concurrently, polar flagellar genes are derepressed, enhancing the bacterial swimming motility in liquid. Thus, LafI functions as a central hub that integrates environmental cues to precisely coordinate *V. parahaemolyticus* motility and virulence through a regulatory circuit.

The synthesis of flagella and virulence factors consumes a substantial percentage of the cellular protein budget. As a result, it is under the stringent control of multiple regulators and environmental conditions (35, 36). Such regulatory precision is especially crucial for pathogens. Here, we unveiled a previously uncharacterized transcription factor, LafI, that coordinates motility and virulence via a regulatory circuit between the lateral flagellar systems and T3SS1 in *V. parahaemolyticus* (Fig. 6). These findings advance our understanding of *V. parahaemolyticus* pathogenesis.

Bacterial motility facilitates survival and proliferation in natural environments, a capability that also underpins the pathogenicity of numerous infectious bacteria (35, 37). Our investigation into the swarming deficiency of *V. parahaemolyticus* clinical strain HZ revealed an unconventional regulatory mechanism controlling this motility phenotype. Despite encoding a complete lateral flagellar gene cluster, the HZ strain exhibited transcriptional repression of lateral flagellar genes, leading to impaired swarmer cell differentiation and lateral flagellum synthesis under surface conditions (Fig. 1). This parallels the observations in *Salmonella* strains with dysregulated flagellar regulators, leading to motility defects (38), which highlights the critical role of transcriptional regulation in coordinating bacterial motility. The discovery of LafI as a key regulator of the flagellar genes promotes our understanding of motility regulation in *V. parahaemolyticus*. LafI harbors a conserved HTH_LUXR (SM00421) domain, which is characteristic of LuxR-type regulators. These regulators are ubiquitously distributed across diverse microbial taxa and function as global activators that control quorum-sensing circuits, virulence factor production, and central metabolic pathways (30, 39, 40). However, our transcriptomic data suggested that LafI did not integrate into quorum-sensing circuits in *V. parahaemolyticus*, while it acts as a negative regulator by repressing the expression of both polar and lateral flagellar genes. When *lafI* was deleted in the HZ strain, we observed a restoration of swarming and an increase in elongated swarmer-like cells; meanwhile, deletion of *opaR*, the master quorum-sensing regulator, had no observable impact on the bacterial swarming behavior (Fig. 2). These results suggested that LafI represents a non-canonical LuxR-type regulator that orchestrates motility programs independently of quorum-sensing pathways in *V. parahaemolyticus*.

Motility has usually been associated with enhanced virulence in many bacterial pathogens, as it allows bacteria to reach and colonize host tissues more effectively (10, 41–43). However, we found that some *V. parahaemolyticus* clinical isolates, like HZ strain, display swarming deficiency *in vitro* while retaining pathogenicity *in vivo*, implying the existence of uncharacterized pathogenicity mechanisms that coordinate swarming motility and host adaptation in these *V. parahaemolyticus* strains. Here, we found that the function of LafI extends far beyond motility and works as an important regulator in *V. parahaemolyticus* under both liquid and surface conditions (Fig. 3 and 4). We demonstrated that LafI inhibits the T3SS1 cytotoxic arsenal via a LafK^L^-ExsA regulatory cascade in liquid conditions. LafK^L^ is a σ^54^-dependent regulator for the lateral flagellar genes and thus contributes to *V. parahaemolyticus* swarming motility (31). ExsA is the master T3SS1 regulator, which has been proven to activate the transcription of the majority of T3SS1 genes (44, 45). While our transcriptomic and genetic data clearly establish LafI-mediated repression of T3SS1 in liquid, the regulatory outcome differs on solid surfaces. We propose that this condition-specificity may be explained by the presence of additional, surface-specific regulatory factors that modulate T3SS1 expression. In the complex signaling environment on a surface, these other factors might override or compensate for the inhibitory function of LafI, rendering its regulatory effect less pronounced or redundant under those specific conditions. A previous study showed that swarming and T3SS1 gene expression were linked by LafK^L^ and additionally connected by a negative-feedback loop on the swarming regulon propagated by ExsA (34). However, our study revealed that ExsA contributes to the expression of the lateral flagellar genes under the *lafI*-deleted background on surface conditions, which suggests a regulatory circuit between T3SS1 and the flagellar system with a positive-feedback loop. We speculate that the discrepancy stems from the strain-specific genetic backgrounds of the *V. parahaemolyticus* isolates used in these studies, coupled with their distinct responses to a key environmental cue. Such a regulatory circuit enables LafI to integrate multifactorial environmental signals and function as a central virulence coordinator in *V. parahaemolyticus*. By dually regulating both the lateral flagella and the T3SS1 cytotoxicity, LafI may couple the classical motility-virulence paradigm in response to the perceived environmental cues, thereby enhancing bacterial ecological adaptability and survival advantage within the host.

In addition to the C-terminal DNA-binding domain, HTH_LUXR, many multiple-domain LuxR regulators usually include signal-binding domains at the N-terminus, such as REC, PAS, AAA, FHA, and CHD domains (30). Here, we noticed that LafI contains a REC domain at the N-terminus (Fig. 2A), which is commonly found in most response regulators of two-component systems (46). A typical two-component system is composed of an additional histidine kinase, which works as a transmembrane sensor (46). Protein-protein interaction (PPI) network analysis showed that a sensor histidine kinase protein, Q4437_14250, may interact with LafI (Fig. S5A). Further analysis revealed that, despite high sequence similarity of LafI and Q4437_14250 to the acetate-metabolism two-component system CrbRS of *V. cholerae* (47, 48), the former pair functionally diverged in regulating motility (Fig. S5B and C), suggesting they may not form a typical two-component system analogous to CrbRS. We also attempted to investigate direct DNA binding of LafI to the promoter regions of its putative target genes (*lafK^L^* and *exsA*) using electrophoretic mobility shift assays (EMSA) (Fig. S6). Despite multiple attempts under various experimental conditions, we were unable to obtain consistent positive results. However, our genetic evidence indicates that LafI relies on its HTH_LUXR domain to regulate the expression of *lafK^L^* and *exsA*, as well as to control swarming motility in *V. parahaemolyticus* (Fig. S7). The lack of detectable DNA binding in EMSA may be attributable to technical limitations. For instance, the recombinant LafI protein may not have been in its native active conformation or might need some specific signals to be active; as a LuxR-type regulator with a REC domain, LafI might require post-translational modifications, such as phosphorylation, for optimal DNA-binding activity (30). LuxR-type regulators typically recognize conserved *lux* box sequences (49, 50). However, our bioinformatic analysis revealed no canonical *lux* boxes in the promoter regions of LafI-regulated genes. Interestingly, these promoters contain extended AT-rich sequences, well-established binding sites for the global silencer H-NS, which preferentially targets curved AT-rich DNA to repress horizontally acquired genes and virulence factors in enteric bacteria (51, 52). In *V. parahaemolyticus*, H-NS directly binds AT-rich promoters of multiple virulence genes, including those within the T3SS1 and T3SS2 clusters (44, 52–54). However, our transcriptomic data show that *hns* transcript levels are unchanged in the Δ*lafI* mutant under either liquid or surface conditions. Moreover, swarming motility and *exsA* promoter activity in the Δ*lafI*Δ*h-ns* strain were significantly increased compared to those in the Δ*h-ns* strain (Fig. S8), suggesting that LafI may function independently of H-NS in regulating these phenotypes. Our future work will be focused on establishing a robust transposon mutagenesis system in *V. parahaemolyticus* to identify additional components and potential interaction partners of the LafI regulatory network through unbiased genome-wide screening.

The function of regulatory factors can vary significantly across different bacterial isolates, often reflecting adaptation to specific ecological niches. For example, EnvZ/OmpR regulates the expression of porin genes in response to medium osmolarity in *Escherichia coli*, while they directly sense host ferric iron and mediate the virulence of *V. parahaemolyticus* (55, 56). To estimate the potential role of LafI among different *V. parahaemolyticus* isolates, we screened the NCBI database for LafI orthologues and performed the genetic evolutionary analyses, which showed that LafI is ubiquitous and highly conserved among diverse *V. parahaemolyticus* isolates, regardless of clinical or environmental origin (Fig. S9A). To determine if LafI’s regulatory role exhibits isolate dependency, we further generated *lafI* deletion mutants in two distinct *V. parahaemolyticus* strains, the swarming-proficient clinical strain RIMD and the swarming-deficient environmental isolate SD33 (57). We observed that LafI had no detectable effect on swarming motility in RIMD, whereas it remained functional in SD33, similar to its role in the HZ strain (Fig. S9B and C). Further transcription analyses revealed that mRNA levels of *lafI* were significantly higher in the HZ and SD33 strains than in the RIMD strain (Fig. S9D), which may partially explain the different role LafI plays in RIMD. These results suggested that LafI exhibits isolate-dependent activity patterns that correlate with expression-level variations, potentially reflecting evolutionary adaptation to diverse ecological niches.

In summary, this study unveiled that LafI functions as an important regulator that integrates motility and virulence in *V. parahaemolyticus* through a regulatory circuit between the lateral flagellar systems and T3SS1. The discovery of LafI provides new insight into the motility-virulence trade-offs in *V. parahaemolyticus* and exemplifies how pathogens may exploit regulatory circuits to fine-tune their behavior to complex environments. Future studies will explore the environmental signals or the upstream regulatory factors that modulate LafI activity.

## MATERIALS AND METHODS

### Bacterial strains, plasmids, and culture conditions

The bacterial strains and plasmids used in this study are listed and described in Table S3. The *V. parahaemolyticus* strains were streptomycin-resistant and grown in modified Luria-Bertani (MLB) broth with 3% NaCl at 37°C. *Escherichia coli* strains DH5α and BL21 (DE3) were grown in Luria-Bertani (LB) broth at 37°C. When required, the culture medium was supplemented with 50 μg/mL streptomycin, 5 μg/mL chloromycetin, 10% (wt/vol) sucrose, 0.1 mM isopropyl β-D-1-thiogalactopyranoside (IPTG), or 0.02% (wt/vol) arabinose.

Growth of *V. parahaemolyticus* strains was measured by recording the values of optical density at 600 nm (OD_600_) in triplicate. Briefly, the strains grown in the logarithmic phase were diluted to an OD_600_ of 0.01 in MLB, which was incubated statically at 37°C. The value of OD_600_ was determined at 1-h intervals for 12 h using the microplate reader (Bio-Tek).

### Bioinformatics analysis

The comparative genomic analysis between *V. parahaemolyticus* strains HZ and RIMD was carried out by using the Mauve alignment system (58). The promoter region of each target operon was predicted by the BProm program (SoftBerry). The SMART database (https://smart.embl.de/) was used for the analysis of protein domain architectures (59). Amino acid sequences used in this study were obtained from the NCBI database (http://blast.ncbi.nlm.nih.gov/). The similarity between amino acid sequences was analyzed by the Basic Local Alignment Search Tool. The phylogenetic trees were constructed by the MEGA7 software based on the amino acid sequences of LafI orthologs using the neighbor-joining method. The PPI network was analyzed by using STRING (https://cn.string-db.org/).

### Recombinant DNA techniques

The suicide plasmid pDS132 was used to make the in-frame markerless deletion strain of *V. parahaemolyticus* as described previously (22). Briefly, the upstream and downstream homology arms of the target gene were assembled into the pDS132 plasmid in the *E. coli* DH5α strain. The recombinant pDS132 plasmid was further transferred into *V. parahaemolyticus* by *E. coli* DH5α λpir using a conjugation method. A putative deletion mutant of *V. parahaemolyticus* was identified using PCR and verified by sequencing.

The reporter plasmid pBBR-*lux* was used to identify the activity of the target gene promoter as described previously (22). The predicted promoter region was amplified by PCR using specific primers and assembled into the pBBR-*lux* plasmid. The pBAD24 (containing an arabinose-inducible promoter) and pMAL (containing an IPTG-inducible promoter) plasmids were used to overexpress the proteins LafI and ScrABC in *V. parahaemolyticus*. The pColdII plasmid was used to express the recombinant LafI protein in *E. coli*. The open reading frames of these genes were amplified by PCR using specific primers and further inserted into these plasmids. These recombinant plasmids were transferred into *E. coli* strain for amplification and verified by sequencing. The primers used for PCR amplification were designed by Clone Manager 9 and listed in Table S4. All enzymes used in this study were purchased from New England BioLabs and APExBIO.

### Transmission electron microscopy

TEM was utilized to analyze the morphological changes of *V. parahaemolyticus* strain under liquid or surface conditions. For the liquid condition, the strains were grown to the logarithmic phase in MLB at 37°C and collected by centrifugation. For the surface condition, the strains were collected from the swarming plate that was incubated at 37°C for 8 h. The strains were then suspended in 50 μL monoethanolamine buffer and applied to carbon-coated copper grids and stood for 2 min at room temperature. Excess liquid was removed from the samples, and the strains were stained with 10 μL of 0.5% phosphotungstic acid. The H-7650 system (Hitachi) was applied to examine the dried grids according to the manufacturer’s instructions.

### RNA extraction, RT-qPCR, and transcriptome sequencing

*V. parahaemolyticus* was collected and rinsed thrice with ice-cold phosphate-buffered saline (PBS). For the liquid condition, the strains were grown to the logarithmic phase in MLB at 37°C and collected by centrifugation (5,000 × *g*, 10 min, 4°C). For the surface condition, the strains were collected from the swarming plate that was incubated at 37°C for 8 h. Total RNA was isolated using TRIzol reagent (Vazyme, China) following the manufacturer’s protocol. RNA purity and concentration were verified spectrophotometrically (A260/A280 ratio ≥1.8; NanoDrop 2000, Thermo Fisher Scientific). Residual genomic DNA was eliminated, and cDNA was synthesized from the RNA using DNase I (HiScript II 1st Strand cDNA Synthesis Kit, Vazyme). Gene expression analysis was conducted on an Mx3000P system (Agilent Technologies) with ChamQ Universal SYBR qPCR Master Mix (Vazyme). The 16S rRNA gene served as the endogenous control for normalization (22, 60). Primer sequences are provided in Table S4. Relative transcript levels were calculated using the 2^−ΔΔCT^ method (61). All reactions were performed in triplicate. Transcriptomic libraries were prepared using the TruSeq Stranded Total RNA Library Prep Kit (Illumina) and sequenced on an Illumina HiSeq 2000 platform. Briefly, RNA was fragmented (60–200 nt), reverse-transcribed into double-stranded cDNA, and ligated with Illumina adapters. Raw reads were filtered to remove low-quality sequences and aligned to the *V. parahaemolyticus* reference genome using HISAT2. Differential gene expression (DEG) was identified via DESeq2 (v1.6.3) with thresholds of |log2 fold change| ≥ 1 and adjusted *P* value (*q*) < 0.05. Functional enrichment of DEGs was analyzed through Gene Ontology (GO) databases using Goatools.

### Motility assays

Swarming plates were prepared by combining 2.5% (wt/vol) brain heart infusion (BHI) broth, 1.2% (wt/vol) agar, 1.5% (wt/vol) NaCl, and 50 μg/mL streptomycin (62). Swimming plates contained 2.5% BHI broth, 0.25% (wt/vol) agar, 1.5% (wt/vol) NaCl, and 50 μg/mL streptomycin (62). For strains harboring the pBAD24 or pMAL plasmids, media were supplemented with 5 μg/mL chloramphenicol, 0.02% (wt/vol) arabinose, or 0.1 mM IPTG. Freshly poured plates were air-dried for 3 h at 25°C, and surface bubbles were eliminated using a sterile pipette tip. A single isolated colony of the strain of interest from MLB agar was transferred to the swarming plates or to the center of the swarming plates using an aseptically handled toothpick. Swarming and swimming motility phenotypes were assessed by incubating respective agar plates at 37°C for 12 and 4 h, followed by quantitative analysis of motility zones. The motility areas were photographed, and the diameter of the digital images was quantified via ImageJ software (v1.53). Each sample procedure was repeated at least three times.

### Bioluminescence reporter assay

Bacterial cultures carrying recombinant bioluminescence reporter plasmids were diluted 1:100 in fresh MLB and grown statically at 37°C for 7 h. For the strains subcultured on swarming plates, swarming plates were inoculated with 10 μL of pre-cultured cells and incubated at 37°C for 8 h. The strains were harvested from plate surfaces and resuspended in sterile PBS. Luminescence intensity and optical density (OD_600_) were quantified using a Bio-Tek Synergy HT spectrophotometer. Luminescence expression was calculated as the luminescence per unit of OD_600_. All experiments included at least three biological replicates.

### Cytotoxicity assay

HeLa cells were maintained in DMEM (Gibco) containing 10% fetal bovine serum (FBS; Gibco) at 37°C under 5% CO₂. The assay was performed as reported previously, with some modifications (63). For cytotoxicity assays, confluent monolayers in 96-well plates were rinsed three times with pre-warmed PBS and infected with bacterial suspensions at a multiplicity of infection of 10 for 2 h. LDH release was quantified using the LDH Cytotoxicity Assay Kit (Beyotime) according to the manufacturer’s protocol. Absorbance at 490 nm was measured on a Bio-Tek Synergy HT spectrophotometer. Percent cytotoxicity was calculated for infected cultures after subtraction of the background LDH release values from uninfected HeLa cells. All experiments included at least three biological replicates.

### Zebrafish infection assay

Sexually mature zebrafish (5 months old) were acclimatized in recirculating aquaria (25°C ± 1°C, 12-h light/dark cycle) for 7 days prior to infection. For survival assays, fish were terminally anesthetized with 1% ethyl 3-aminobenzoate methane sulfonate (Sigma-Aldrich) and intraperitoneally administered 20 μL of bacterial suspensions (2.0 × 10^7^ CFU per fish; *n* = 15) or sterile PBS (*n* = 15, control group). Mortality was recorded at 12-h intervals over a 72-h period post-infection.

### Electrophoretic mobility shift assay

DNA-binding activity of LafI was evaluated by EMSA (22). Briefly, the *lafI* gene was cloned into the pColdII vector and expressed in *E. coli* BL21(DE3). The protein expression was induced with 0.1 mM IPTG at 18°C for 10 h. The bacterial cells were harvested by centrifugation and lysed by sonication, followed by purification of the recombinant LafI protein using a Ni-nitrilotriacetic acid spin column according to the manufacturer’s protocol (GE Healthcare). The recombinant LafI protein was verified by SDS-PAGE. The target promoter regions were amplified by PCR and purified with a gel extraction kit (Vazyme) to generate DNA probes. Fixed amounts of DNA probes were incubated with increasing concentrations of LafI protein in binding buffer at 37°C for 30 min (22). Protein-DNA complexes were resolved on 6% polyacrylamide gels in 0.5× TBE buffer at 200 V for 45 min under cold conditions. Nucleic acids were visualized by staining with SYBR Gold nucleic acid stain (Invitrogen), and the images were captured.

### Statistical analysis

Data were presented as mean ± standard deviation (SD). Statistical analyses were conducted using an unpaired two-tailed Student’s *t*-test, a one-way ANOVA followed by Tukey’s test, or the log-rank (Mantel-Cox) test with the GraphPad software package. For all tests, a *P* value < 0.05 was considered statistically significant.

## Data Availability

The RNA-seq data have been deposited in NCBI under BioProject no. PRJNA1247182 and PRJNA1247884. The gene designated *motI* in the BioProject records corresponds to *lafI* described in this study; the gene name was revised during the peer review process.

## SUPPLEMENTAL MATERIAL

Supplemental figures aem.01123-26-s0001.pdf Fig. S1 to S9.

Table S1 aem.01123-26-s0002.xlsx Differentially expressed genes in the Δ*lafI* strain versus the wild-type HZ strain under the surface condition.

Table S2 aem.01123-26-s0003.xlsx Differentially expressed genes in the Δ*lafI* strain versus the wild-type HZ strain under the liquid condition.

Tables S3 and S4 aem.01123-26-s0004.pdf Table S3, bacterial strains and plasmids; Table S4, primers.

ASM does not own the copyrights to Supplemental Material that may be linked to, or accessed through, an article. The authors have granted ASM a non-exclusive, world-wide license to publish the Supplemental Material files. Please contact the corresponding author directly for reuse.
