# Supplementary material for: The transcription factor LafI integrates control of flagellar motility and T3SS1-mediated virulence in Vibrio parahaemolyticus
Source: Appl Environ Microbiol. 2026 Jul 29;92(8):e01123-26. doi: 10.1128/aem.01123-26 (PMC13488335; doi:10.1128/aem.01123-26)
Supplement: Supplementary file 1 [file aem.01123-26-s0001.pdf]

Supplementary Figures S1 to S9

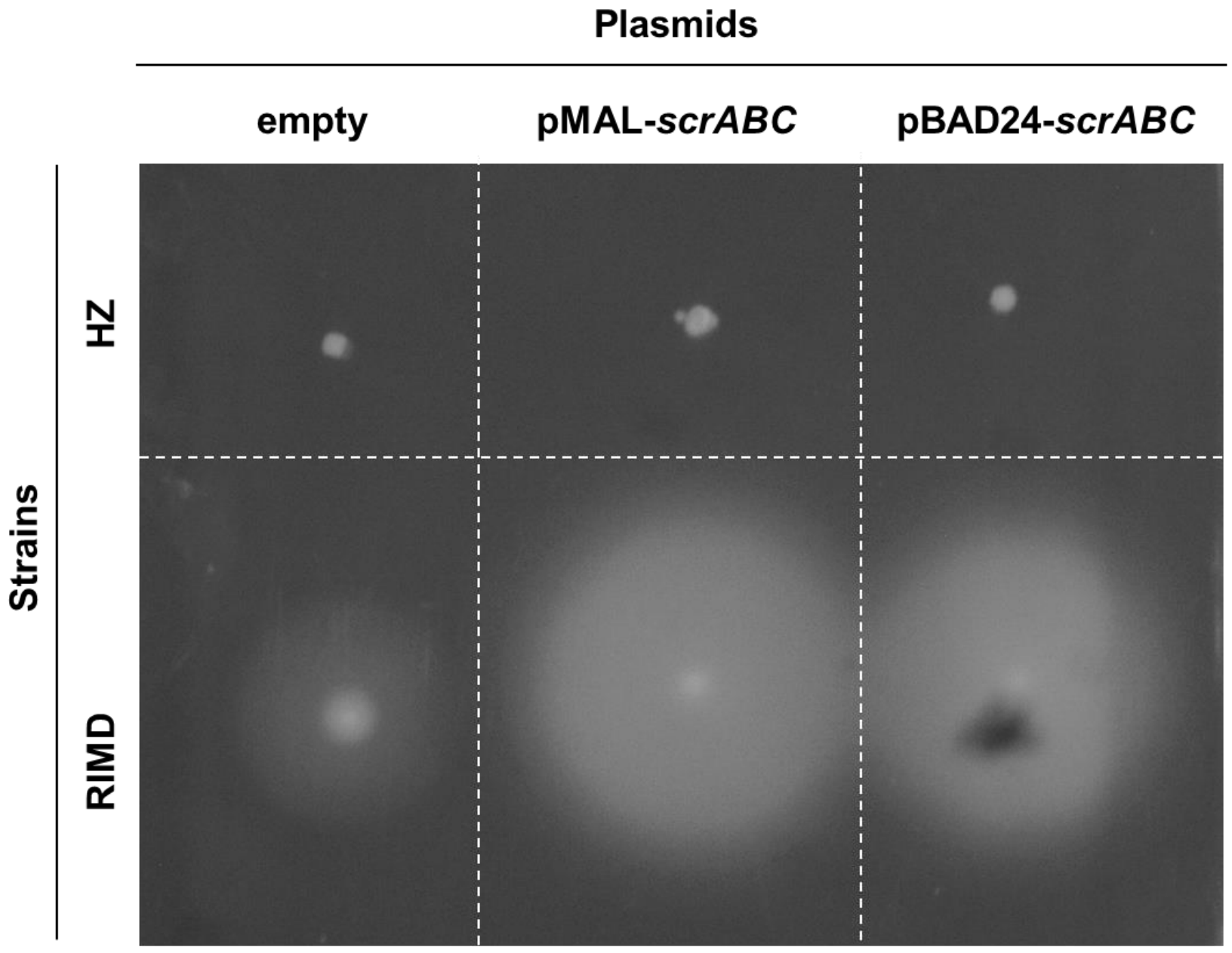

Figure S1: Overexpressing the *scrABC* operon doesn't recover the swarming defects of the HZ strain. The swarming colony of the indicated strains.

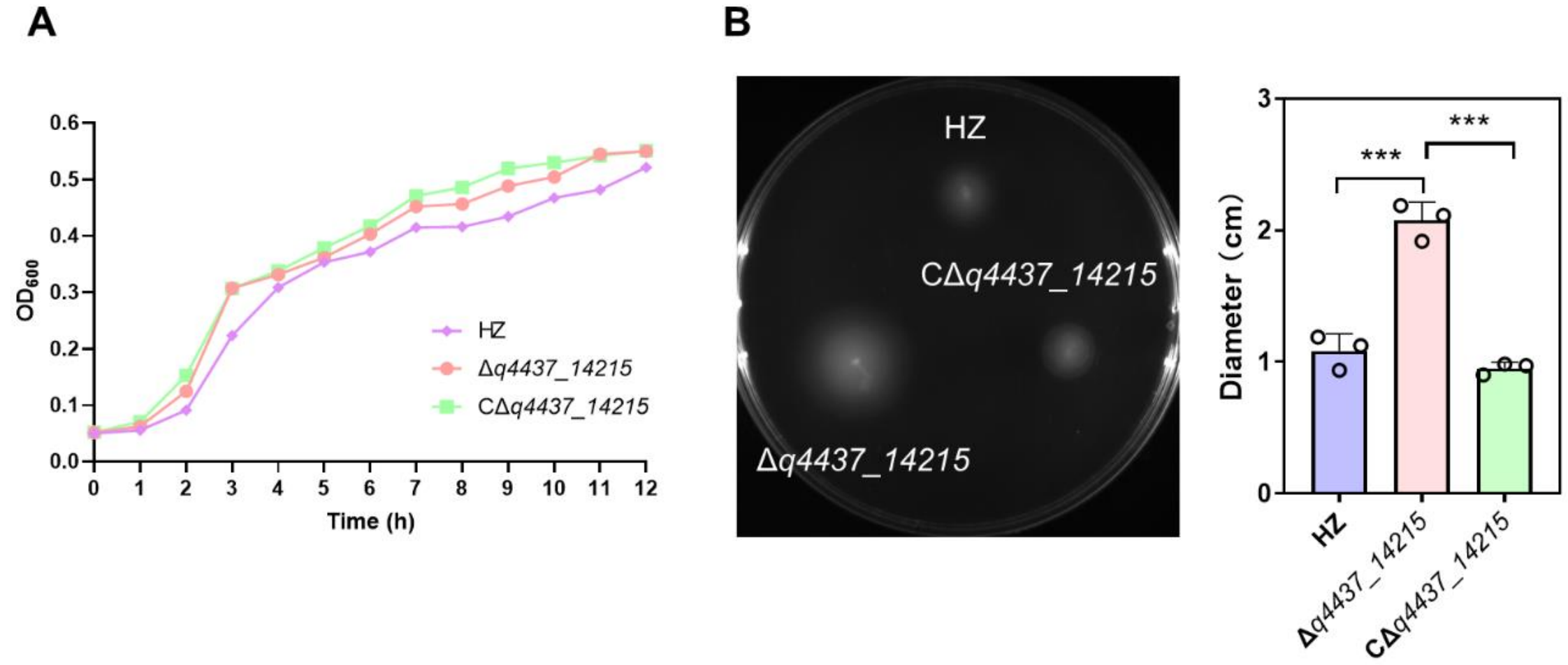

Figure S2: The bacterial growth and swimming of the HZ, Δ*q4437_14215*, and CΔ*q4437_14215* strains. **A** The bacterial growth curves of the HZ, Δ*q4437_14215*, and CΔ*q4437_14215* strains under static conditions. Bacterial cell density was measured and reported as the value of OD_{600}. **B** The swimming colony and the migration diameter of the wild-type HZ, Δ*q4437_14215*, and CΔ*q4437_14215* strains. The migration diameter of the swimming colonies was measured by using ImageJ software. The CΔ*q4437_14215* strain carries a complementary plasmid for *q4437_14215*, while the HZ and Δ*q4437_14215* strains harbor an empty pBAD24 plasmid. The unpaired two-tailed Student's *t*-test was used for statistical analysis (***, $P < 0.001$).

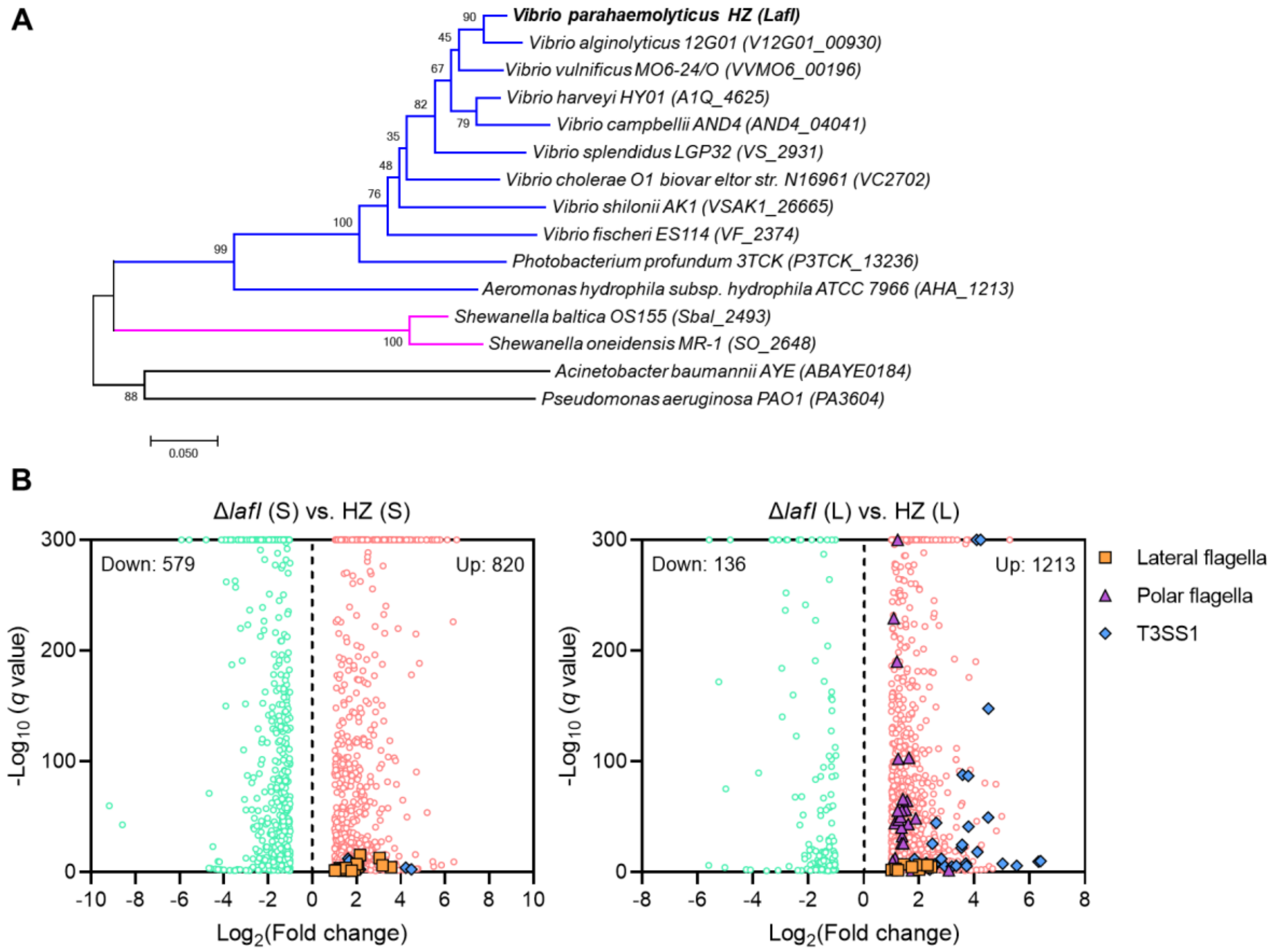

Figure S3: Identification of LafI-regulated genes. A Phylogenetic analysis of LafI homologs in prokaryotes. The phylogenetic tree was constructed with the MEGA7 software using the neighbor-joining method. **B-C** Volcano plots visualizing differential gene expression between the Δ*lafI* and wild-type HZ strains were generated from RNA-seq data under the surface (S) and liquid (L) conditions. The $\log_2$ (Fold change) and $-\log_{10}$ (q value) are plotted on the *x*- and *y*-axes, respectively. Red dots represent up-regulated genes, while green dots represent down-regulated genes.

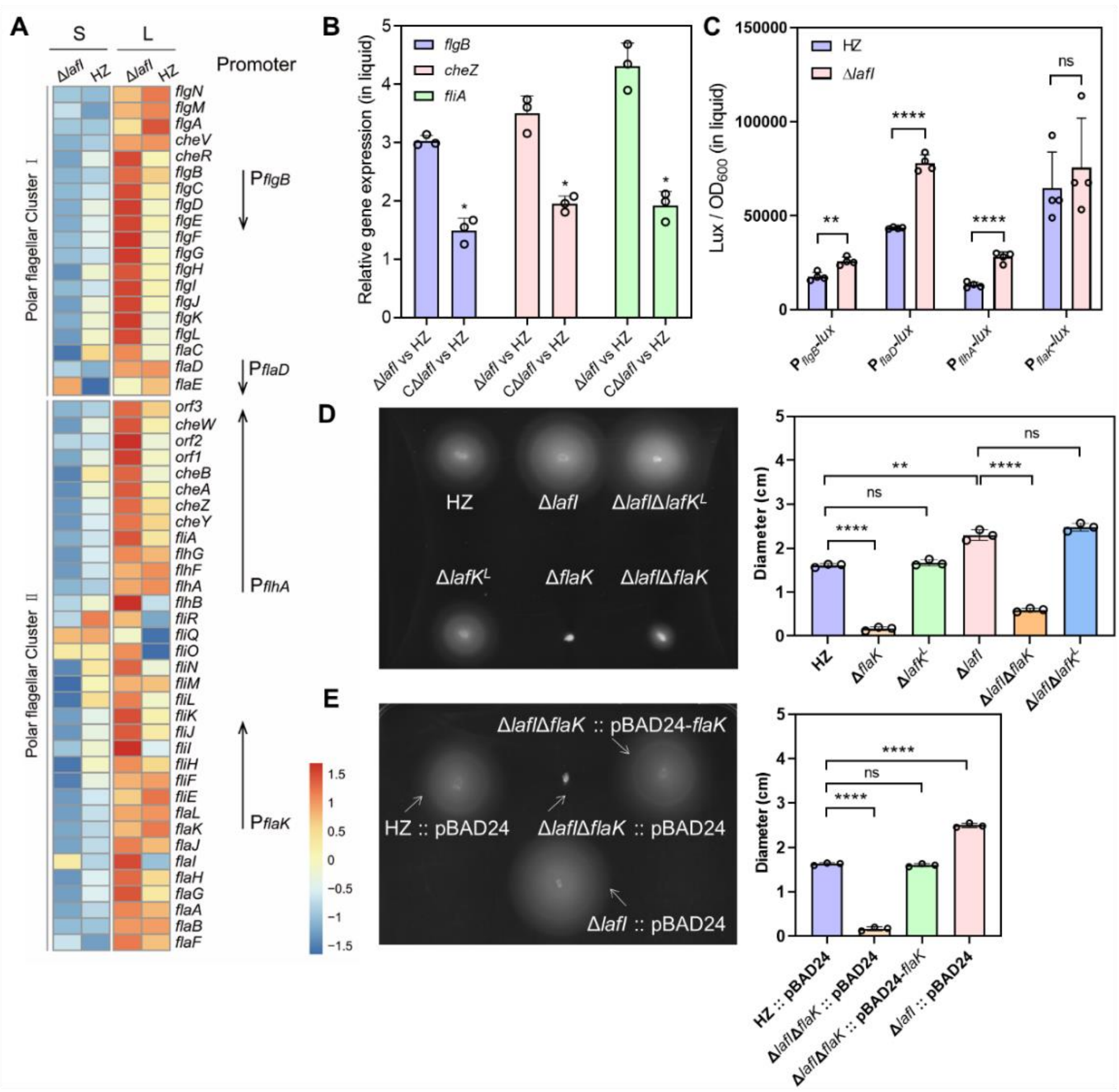

Figure S4: LafI regulates the expression of the polar flagellum. A Heat map analysis of the relative expression level of the polar flagellar gene clusters in the Δ*lafI* and wild-type HZ strains under the surface (S) and liquid (L) conditions. The expression values were Z-score normalized to a range of -1.5 to 1.5. The arrows indicate the transcription direction of the operon. **B** The mRNA levels of the polar flagellar genes in the wild-type HZ, Δ*lafI*, and CΔ*lafI* strains as determined by RT-qPCR. The CΔ*lafI* strain carries a complementary plasmid for *lafI*, while the HZ and Δ*lafI* strains harbor an empty pBAD24 plasmid. The results of RT-qPCR are expressed as means ± SD deviation from three independent experiments. **C** The transcription levels of the polar flagellar gene clusters in the wild-type HZ and Δ*lafI* strains were assessed by measuring luminescence in the strains containing P_{flgB}-*lux*, P_{flaD}-*lux*, P_{flhA}-*lux*, and P_{flaK}-*lux*, respectively. **D-E** The swimming colony and the migration diameter of the wild-type HZ and the indicated mutant strains. The migration diameter of the swimming colonies was measured by using ImageJ software. The unpaired two-tailed Student's *t*-test (**B**, **C**) and the one-way ANOVA followed by Tukey's test (**D, E**) were used for statistical analysis (ns, $P > 0.05$; *, $P < 0.05$; **, $P < 0.01$; ****, $P < 0.0001$).

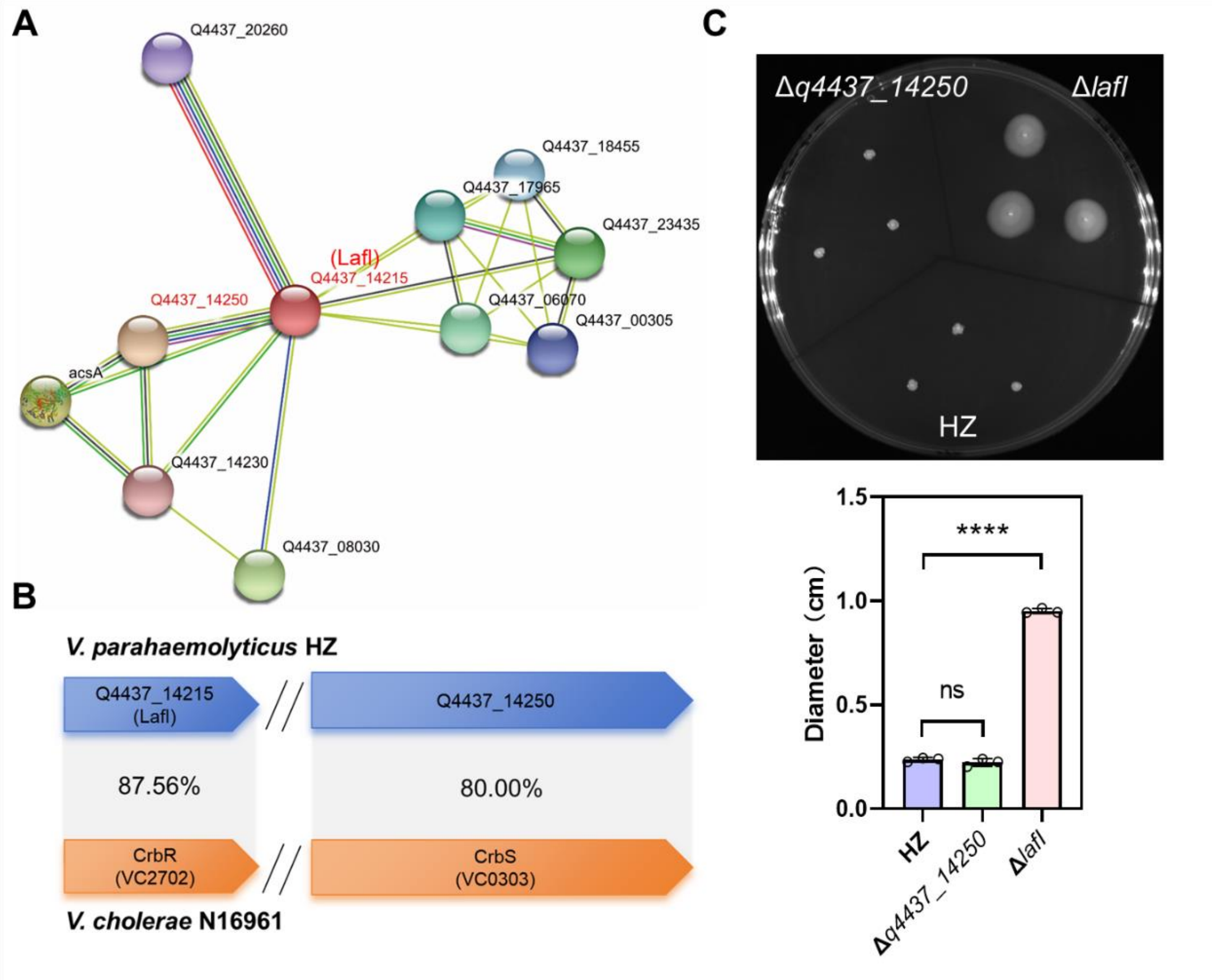

Figure S5: Functional assessment of Q4437_14250. A PPI network analysis of LafI. **B** Homology analyses of amino acid sequences encoded by *lafI* and *q4437_14250* genes using BLAST. The identities of the amino acid sequences of each protein are shown between *V. parahaemolyticus* strain HZ and *V. cholerae* N16961. **C** The swarming colony and the migration diameter of the indicated strains. The migration diameter of the swarming colonies was measured by using ImageJ software. The unpaired two-tailed Student's *t*-test was used for statistical analysis (ns, $P > 0.05$; ****, $P < 0.0001$).

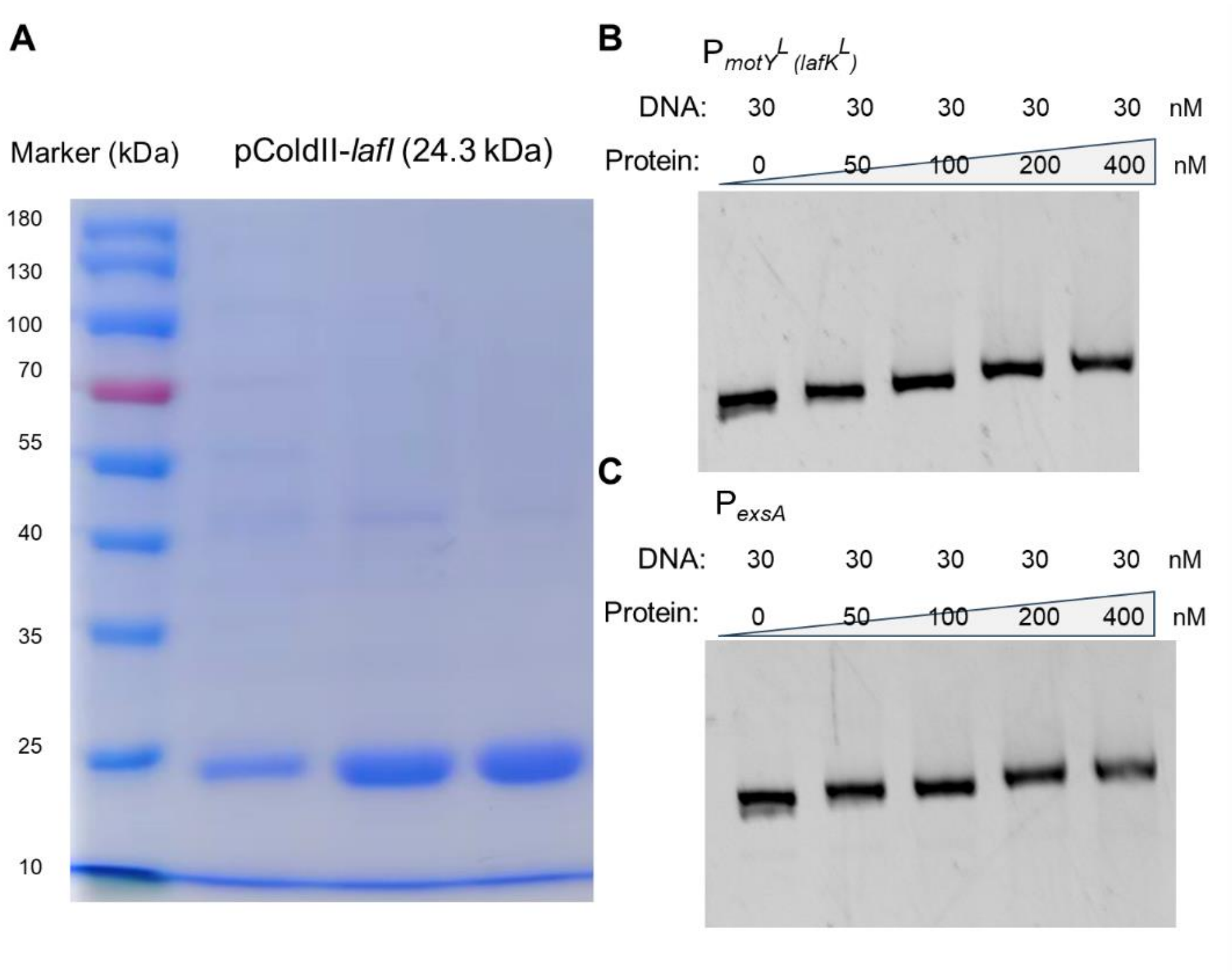

Figure S6: Expression and DNA-binding activity assessment of recombinant LafI. A The recombinant LafI protein was expressed from the pColdII-*lafI* vector. **B-C** EMSA showing that LafI could not bind to the promoter region of the target gene clusters. Each reaction mixture contains DNA probe (30 nM) and LafI protein (0 to 400 nM).

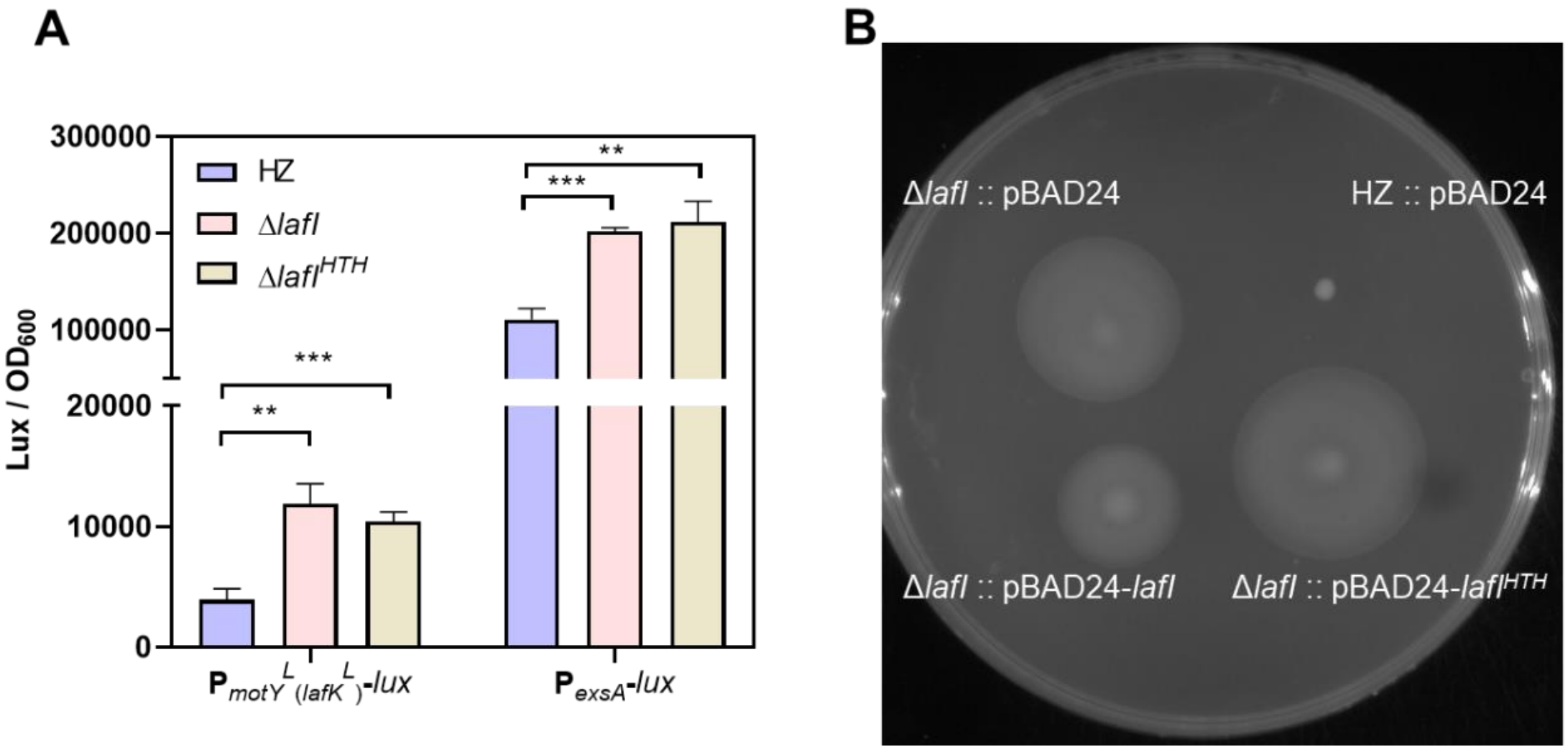

Figure S7: HTH_LUXR domain of LafI is essential for its regulatory function. A The transcription levels of the target genes in the wild-type HZ and the indicated strains were assessed by measuring luminescence in the strains containing P_{motY^L} $_{(lafK^L)}$-*lux*, and P_{exsA}-*lux*, respectively. **B** The swarming colony of the wild-type HZ and the indicated strains. The unpaired two-tailed Student's *t*-test was used for statistical analysis (**, $P < 0.01$; ***, $P < 0.001$).

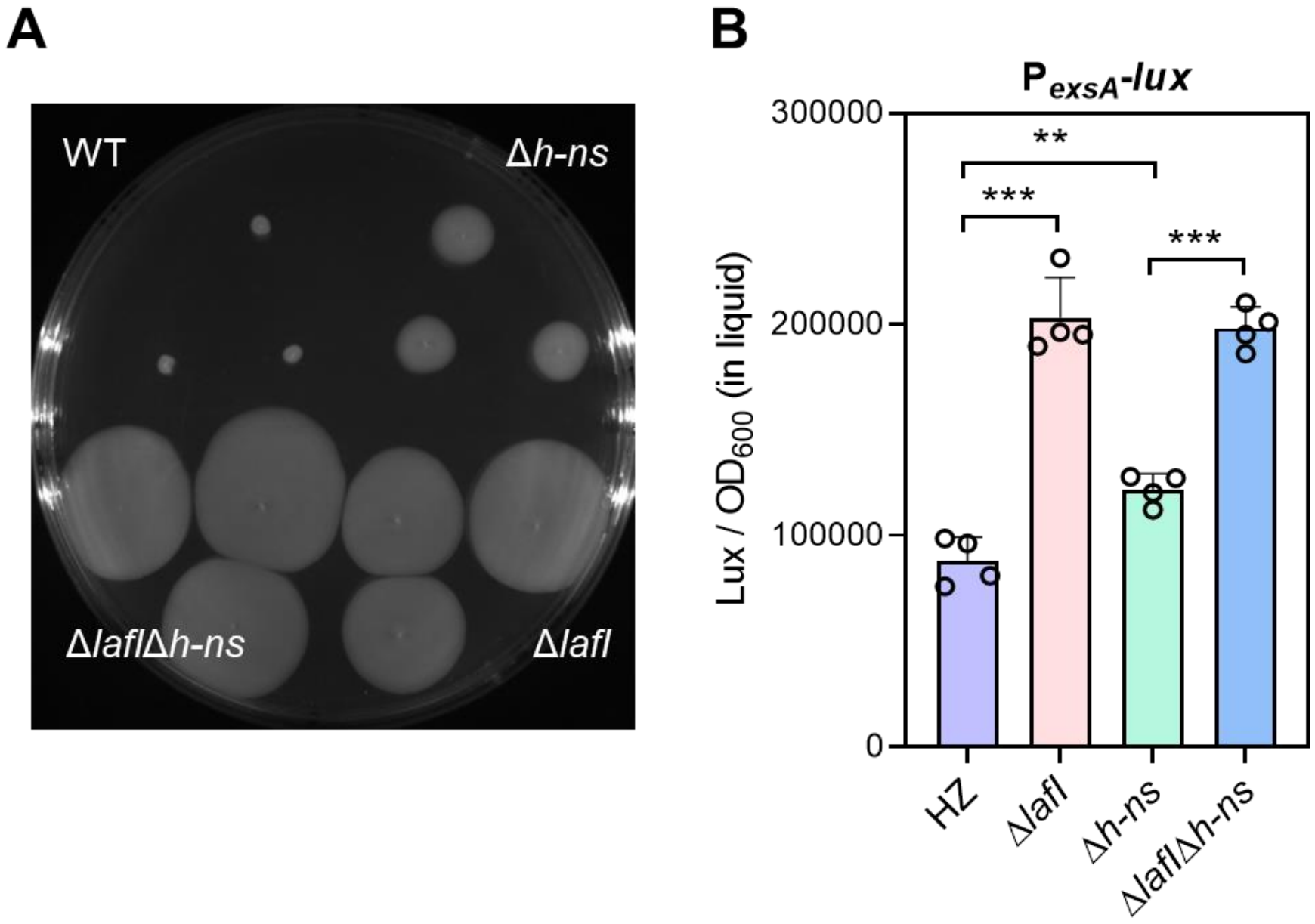

Figure S8: Functional assessment of H-NS. A The swarming colony of the indicated strains. **B** The transcription levels of *exsA* in the wild-type HZ and the indicated mutant strains were assessed by measuring luminescence in the strains containing promoter-*lux* transcriptional fusion plasmid. Bacterial cultures were grown in liquid at 37˚C. The unpaired two-tailed Student's *t*-test was used for statistical analysis (**, $P < 0.01$; ***, $P < 0.001$).

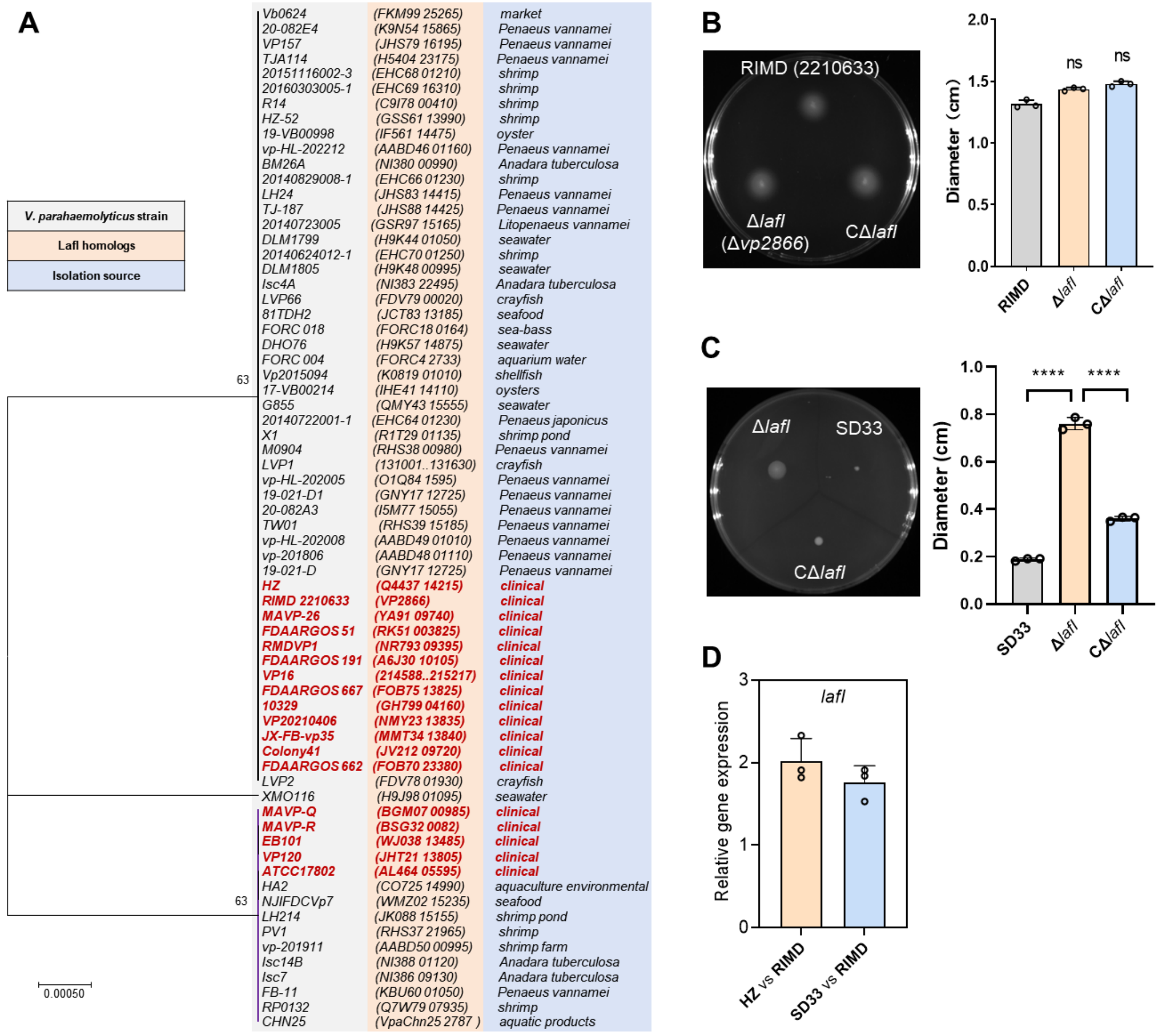

Figure S9: LafI exhibits widespread occurrence among diverse isolates and plays different roles in RIMD and SD33 strains. A Phylogenetic analysis of LafI homologs in different *V. parahaemolyticus* isolates. The phylogenetic tree was constructed with the MEGA7 software using the neighbor-joining method. The red letters highlight the clinical *V. parahaemolyticus* isolates. **B-C** The swarming colony and the migration diameter of the indicated strains. The CΔ*lafI* strain carries a complementary plasmid for *lafI*, while the wild-type and Δ*lafI* strains harbor an empty pBAD24 plasmid. The migration diameter of the swarming colonies was measured by using ImageJ software. **D** The mRNA levels of *lafI* in the wild-type HZ, RIMD, and SD33 strains as determined by RT-qPCR. The results of RT-qPCR are expressed as means ± SD deviation from three independent experiments. The unpaired two-tailed Student's *t*-test was used for statistical analysis (ns, $P > 0.05$; ****, $P < 0.0001$).
