# Supplementary material for: The transcription factor LafI integrates control of flagellar motility and T3SS1-mediated virulence in Vibrio parahaemolyticus
Source: Appl Environ Microbiol. 2026 Jul 29;92(8):e01123-26. doi: 10.1128/aem.01123-26 (PMC13488335; doi:10.1128/aem.01123-26)
Supplement: Supplementary file 2 [file aem.01123-26-s0004.pdf]

Supplementary Tables S3 and S4

Table S3 Bacterial strains and plasmids used in this study

**Strains or plasmids**	**Description**	**Source**
Bacterial strains		
HZ	*V. parahaemolyticus*, clinical isolate	Our laboratory
RIMD 2210633	*V. parahaemolyticus*, clinical isolate	Our laboratory
SD33	*V. parahaemolyticus*, isolated from shellfish	Our laboratory (1)
Δ*q4437_14215* (Δ*lafI*)	Deletion mutant of *q4437_14215* with HZ background	This study
Δ*q4437_13385*	Deletion mutant of *q4437_13385* with HZ background	This study
Δ*q4437_15925*	Deletion mutant of *q4437_15925* with HZ background	This study
Δ*q4437_17725*	Deletion mutant of *q4437_17725* with HZ background	This study
Δ*opaR*	Deletion mutant of *opaR* with HZ background	This study
Δ*q4437_19065*	Deletion mutant of *q4437_19065* with HZ background	This study
Δ*q4437_15870*	Deletion mutant of *q4437_15870* with HZ background	This study
Δ*q4437_18230*	Deletion mutant of *q4437_18230* with HZ background	This study
Δ*q4437_19060*	Deletion mutant of *q4437_19060* with HZ background	This study
Δ*q4437_09830*	Deletion mutant of *q4437_09830* with HZ background	Our laboratory (2)
Δ*q4437_14250*	Deletion mutant of *q4437_14250* with HZ background	This study
Δ*flaK*	Deletion mutant of *flaK* with HZ background	This study
Δ$lafA^L$	Deletion mutant of $lafA^L$ with HZ background	This study
Δ*lafI*Δ*flaK*	Deletion mutant of *lafI* and *flaK* with HZ background	This study
Δ*lafI*Δ*flaK*Δ$lafA^L$	Deletion mutant of *lafI*, $lafA^L$, and *flaK* with HZ background	This study
Δ*exsA*	Deletion mutant of *exsA* with HZ background	This study
Δ*lafI*Δ*exsA*	Deletion mutant of *lafI* and *exsA* with HZ background	This study
Δ$lafK^L$	Deletion mutant of $lafK^L$ with HZ background	This study
Δ*lafI*Δ$lafK^L$	Deletion mutant of *lafI* and $lafK^L$ with HZ background	This study
Δ$lafI^{HTH}$	Deletion mutant of HTH domain of *lafI* with HZ background	This study
Δ*h-ns*	Deletion mutant of *h-ns* with HZ background	This study
Δ*lafI*Δ*h-ns*	Deletion mutant of *lafI* and *h-ns* with HZ background	This study
Δ*lafI* (RIMD 2210633)	Deletion mutant of *lafI* with RIMD 2210633 background	This study
Δ*lafI* (SD33)	Deletion mutant of *lafI* with SD33 background	This study
CΔ*q4437_14215*	Δ*q4437_14215* with the plasmid pBAD24-*lafI*	This study
CΔ*lafI* (SD33)	Δ*lafI* (SD33) with the plasmid pBAD24-*lafI*	This study
CΔ*lafI* (RIMD 2210633)	Δ*lafI* (RIMD 2210633) with the plasmid pBAD24-*lafI*	This study
E. coli DH5α	Cloning host for maintaining the recombinant plasmids	Our laboratory
E. coli DH5α λpir	Mobilization of plasmids into *V. parahaemolyticus*	Our laboratory
E. coli BL21 (DE3)	Host for expressing the recombinant proteins	Our laboratory
Plasmids		
pDS132	Suicide vector for *V. parahaemolyticus* mutagenesis	Our laboratory
pDS132-*lafI*	Derived from pDS132 used to knock out *lafI* in HZ, RIMD 2210633, and SD33	This study

pDS132-*q4437_13385*	Derived from pDS132 used to knock out *q4437_13385*	This study
pDS132-*q4437_15925*	Derived from pDS132 used to knock out *q4437_15925*	This study
pDS132-*q4437_17725*	Derived from pDS132 used to knock out *q4437_17725*	This study
pDS132-*opaR*	Derived from pDS132 used to knock out *opaR*	This study
pDS132-*q4437_19065*	Derived from pDS132 used to knock out *q4437_19065*	This study
pDS132-*q4437_15870*	Derived from pDS132 used to knock out *q4437_15870*	This study
pDS132-*q4437_18230*	Derived from pDS132 used to knock out *q4437_18230*	This study
pDS132-*q4437_19060*	Derived from pDS132 used to knock out *q4437_19060*	This study
pDS132-*q4437_14250*	Derived from pDS132 used to knock out *q4437_14250*	This study
pDS132-*flaK*	Derived from pDS132 used to knock out *flaK*	This study
pDS132-$lafA^{L}$	Derived from pDS132 used to knock out $lafA^{L}$	This study
pDS132-*exsA*	Derived from pDS132 used to knock out *exsA*	This study
pDS132-$lafK^{L}$	Derived from pDS132 used to knock out $lafK^{L}$	This study
pDS132-$lafI^{HTH}$	Derived from pDS132 used to knock out HTH domain of *lafI*	This study
pDS132-*h-ns*	Derived from pDS132 used to knock out *h-ns*	This study
pBAD24	Arabinose-induced expression plasmid	Our laboratory
pBAD24-*scrABC*	Derived from pBAD24 used to overexpress ScrABC protein	Our laboratory (3)
pBAD24-*lafI*	Derived from pBAD24 used to overexpress LafI protein	This study
pBAD24-$lafK^{L}$	Derived from pBAD24 used to overexpress $\mathrm{LafK^{L}}$ protein	This study
pBAD24-*flaK*	Derived from pBAD24 used to overexpress FlaK protein	This study
pBAD24-*exsA*	Derived from pBAD24 used to overexpress ExsA protein	This study
pBAD24- $lafI^{HTH}$	Derived from pBAD24 used to overexpress HTH domain of LafI protein	This study
pMAL	IPTG-induced expression plasmid	Our laboratory
pMAL-*scrABC*	Derived from pMAL used to overexpress ScrABC protein	This study
pBBR-*lux*	The bioluminescence reporter containing *luxCDABE* operon of *Vibrio harveyi*	Our laboratory
$\mathrm{P}_{flgB^{L}}$-*lux*	pBBR-*lux* containing the promoter of $flgB^{L}$	Our laboratory (3)
$\mathrm{P}_{lafA^{L}}$-*lux*	pBBR-*lux* containing the promoter of $lafA^{L}$	Our laboratory (3)
$\mathrm{P}_{fliD^{L}}$-*lux*	pBBR-*lux* containing the promoter of $fliD^{L}$	Our laboratory (3)
$\mathrm{P}_{motY^{L}}$-*lux*	pBBR-*lux* containing the promoter of $motY^{L}$	Our laboratory (3)
$\mathrm{P}_{fliM^{L}}$-*lux*	pBBR-*lux* containing the promoter of $fliM^{L}$	This study
P_{flgB}-*lux*	pBBR-*lux* containing the promoter of *flgB*	This study
P_{flaD}-*lux*	pBBR-*lux* containing the promoter of *flaD*	This study
P_{flhA}-*lux*	pBBR-*lux* containing the promoter of *flhA*	This study
P_{flaK}-*lux*	pBBR-*lux* containing the promoter of *flaK*	This study
P_{exsA}-*lux*	pBBR-*lux* containing the promoter of *exsA*	This study
P_{vopN}-*lux*	pBBR-*lux* containing the promoter of *vopN*	This study
P_{vscN}-*lux*	pBBR-*lux* containing the promoter of *vscN*	This study
pColdII	His-tag expressing vector	Our laboratory
pColdII-*lafI*	pColdII inserted in-frame with the *lafI* gene for expressing LafI	This study

References

1. Zhong X, Pan Z, Mu Y, Zhu Y, Zhang Y, Ma J, Yang M, Yao H. 2022. Characterization and

epidemiological analysis of Vibrio parahaemolyticus isolated from different marine products in East China. Int J Food Microbiol 380:109867.
2. Zhong X, Liu F, Liang T, Lu R, Shi M, Zhou X, Yang M. 2024. The two-component system TtrRS boosts Vibrio parahaemolyticus colonization by exploiting sulfur compounds in host gut. PLoS Pathog 20:e1012410.
3. Liu F, Wang F, Yuan Y, Li X, Zhong X, Yang M. 2023. Quorum sensing signal synthases enhance Vibrio parahaemolyticus swarming motility. Mol Microbiol 120:241-257.

Table S4 Primers used in this study

Primer	Primer sequence (5'–3')
pDS132- *lafI*	P1: GCGTCTAGACGTTGCACAAAGTGGTGA
	P2: GGCTTGCGTGCGGTTTGCATTACGGAAGAGAGGG
	P3: AACCGCACGCAAGCCGTCATCATGTTAACG
	P4: GCGGAGCTCCATTCGATTCTGCATGTG
pDS132- *q4437_13385*	P1: GCGTCTAGACTGATTGTGAGAGTTGGAAG
	P2: CAAGCAATGGCTTGCATACTAATCAGAAATAGCTTTCT
	P3: CAAGCCATTGCTTGGGCCGATCAAAACTTGAT
	P4: GCGGAGCTCTTTTGCTTGTTTCAGCAGCGC
pDS132- *q4437_15925*	P1: GCGTCTAGACTAAACATCAATCCTCGTTTT
	P2: ACCAACAACACCATTACGTGGGCAAAACGTAAT
	P3: TGCCCACGTAATGGTGTTGTTGGTTGTGTTGG
	P4: GCGGAGCTCTCGCTTTCCTCATGAAGGAA
pDS132- *q4437_17725*	P1: GCGTCTAGAACAATCTGCGTGTAAGCATGA
	P2: ATTCAAAACAACTACTGCTATTTGGCCCCA
	P3: CAAATAGCAGTAGTTGTTTTGAATGGAGAGCG
	P4: GCGTCTAGATTATTTTGGGATGACTTCACG
pDS132- *opaR*	P1: GCGTCTAGATCGCGATAGATTTAGGTTCAC
	P2: TCTAGGTAAGCGCTTTGTTGCTTACGTTTAAGAG
	P3: AGCGCTTACCTAGATATGCTTTGCATCTAC
	P4: GCGGAGCTCAATCGACGAGCGCGGCTTCATCA
pDS132- *q4437_19065*	P1: GCGTCTAGAGGGATAGCAAGTACGAGTA
	P2: ATGGAACAGTACAGTTGGGCAAAAAGAAACCT
	P3: TTTTTGCCCAACTGTACTGTTCCATAATGTTTCT
	P4: GCGGAGCTCAAACTACTAAAGGAATGCGA
pDS132- *q4437_15870*	P1: GCGTCTAGACGGTTTACCTTGGCTTGCT
	P2: CAACTCGCAGAACAATTGTTCCTCAACCACCT
	P3: GAGGAACAATTGTTCTGCGAGTTGCGCGTTAA
	P4: GCGTCTAGAACCTCTCCTGCTCATGCA
pDS132- *q4437_18230*	P1: GCGTCTAGATCGCTTGTTGGATGCGAGGA
	P2: AGTTACAATGGCGGCATTGTGTAGTCGGCCGGG
	P3: CTACACAATGCCGCCATTGTAACTGCGGAGAAT
	P4: GCGGAGCTCCATCGCGTTTCTCTACCTGA
pDS132- *q4437_19060*	P1: GCGGAGCTCTTACATGCAGTTATTCCTAGTAC
	P2: ACTACAAGGAATTCAGATCACTCTATAGAGATG
	P3: AGAGTGATCTGAATTCCTTGTAGTTTGATGCT

	P4: GCGGAGCTCGATTGAAGCGGCACGCGCAG
pDS132- *q4437_14250*	P1: GCTCTAGAACTCATTTGATGGACGCCATGCCAATCACTT
	P2: CTATTTATAATCTTTATTGCCAAACCCGTT
	P3: TTTGGCAATAAAGATTATAAATAGGGCTCCCA
	P4: GCTCTAGATGTTGGATTACGGTCGCACGCGTTTGTGGGG
pDS132-*flaK*	P1: AACTGCAGTTTACCGGGCGGAAAAAGATGTCGATTTGC
	P2: CTGCTTGTAATTAAAATGCGCAAGTATAATTTGCA
	P3: CTTGCGCATTTTAATTACAAGCAGCTTTGC
	P4: ACATGCATGCCAAGAGCTGGAAAAAGTTGACTTTAATACT
pDS132-*lafA*^{L}	P1: GCTCTAGAACATACAACACGCTTGCCAAGCCATTCGTG
	P2: AGACATTGCCATACTAGGTGCGTAGTTAGTGTGCAT
	P3: ATGCACACTAACTACGCACCTAGTATGGCAATGTCT
	P4: CGAGCTCTTGAGAGATGGAGGTGGAGTTTTTAATGAT
pDS132-*exsA*	P1: GCTCTAGATACTTGTGACTGCTGCTCGTTCAGTG
	P2: CTCATATTGTTGTCTCTTACTTTACTCAAAGCTATCG
	P3: TAAAGTAAGAGACAACAATATGAGATTGGTCTGAGTG
	P4: GCGGAGCTCTCAAGCTGGGAAAGGAGAAATGAAAGTG
pDS132-*lafK*^{L}	P1: GCTCTAGATGAGCTGTCTTCTTTCAGCG
	P2: ACGAATATTTTGTTGGTTCAAAGTCCAGCTTTGGT
	P3: GACCAAAGCTGGACTTTGAACCAACAAAATATTCGTTTTCG
	P4: CGAGCTCATGGAAATGTAAGATATACTTTT
pDS132-*lafI*^{HTH}	P1: ACCGCATGCGATATCGAGCTCCATGCTCGACTTCTACGCCG
	P2: GGAGGACTAAGTCCTATAAATCATTACACGCAGCGTT
	P3: TGCGTGTAATGATTTATAGGACTTAGTCCTCCTCTT
	P4: GTGGAATTCCCGGGAGAGCTCTACGCCGTCGAACTTGCTG
pDS132-*h-ns*	P1: GCTCTAGATAATGAAACAGGAGAATGAGAAAAATCTAT
	P2: AATTGCAGAAGGCTCACGAGAAAATGCACG
	P3: TTTTCTCGTGAGCCTTCTGCAATTCAAGAG
	P4: CGAGCTCCGAAGGTCAAATGAACCAGCTTCATCCAGA
pBAD24- *q4437_14215*	P1: GCGGGTACCTTCATTTAACGAAATAGTCAAAAG
	P2: GCGAAGCTTCTAATCATTCATTTCCGTTAACA
pMAL-*scrABC*	P1: CAACAAGGACCATAGCATATGTATGGCTAATTTCCGACAAGGAT
	P2: TGCCTGCAGGTCGACTCTAGATTACTTATCATCATCATCCTTATAATCTGACCAAGTAGGTTGGTTTAGAAGTT
pBAD24-*lafK*^{L}	P1: GGGGTACCAATGATGACGAAAACGAATATTTTGTTGGT
	P2: GCTCTAGATTAGGCAGCCGAGCCTAGAGCCGACTGTAA
pBAD24-*flaK*	P1: ATCCATAAGATTAGCGGATCCGTCGGCATTTAATAATCAAAACAATG
	P2: TCCGCCAAAACAGCCAAGCTTCTAACGCTGCAAATTATACTTGCG
pBAD24-*exsA*	P1: ATCCATAAGATTAGCGGATCCTCGGTTACATTTAATTAGCGCTATC
	P2: TGCCTGCAGGTCGACTCTAGATTACTTATCATCATCATCCTTATAATCATTCGCGATGGCGACTTGCT
pBAD24-*lafI*^{HTH}	P1: GGTACCCGGGGATCCTCTAGAAGCAGAGAAGATTGCCGCG

	P2: TGCCTGCAGGTCGACTCTAGACTAATCATTCATTTCCGTTAACATGA
P_{fliM^L}-*lux*	P1: CGAGCTCTTAATTACCTATTCTGAAATACTTAAATTG
	P2: CGGGATCCTTATATTTTACCGTGCGTTAACAAACTAAG
P_{flgB}-*lux*	P1: CGAGCTCCGGTATCTCTTATTTGAAAACAAAGCCTGA
	P2: CGGGATCCGTTTGCCTCTACAATTAAGAACTGACCGTT
P_{flaD}-*lux*	P1: CGAGCTCTTTGGCCCTCTCGGCTCAATTTGTAGAAAC
	P2: CGGGATCCGGTGATTTCTCCAATTGATTTTCCGATGTA
P_{flhA}-*lux*	P1: CGGGATCCACTGCGAGGTATCTCAATCGAATTGAAGCG
	P2: CGAGCTCCCCGCCGGAACATCGTCATTAATCGTACAA
P_{flaK}-*lux*	P1: CGGGATCCAAGTAAGAATGATTGCCTTTATTTTAGGTG
	P2: GACTAGTTAATCAAAACAATGCTTTAGGATCGACTTA
P_{exsA}-*lux*	P1: CGAGCTCGTTTAGTTGGCCTGACACAT
	P2: CGCGGATCCCGGTTACATTTAATTAGCGCTATC
P_{vopN}-*lux*	P1: GCGGAGCTCGGATCAAACGCGTTTGTTTG
	P2: GCGGGATCCTCACTTGCACTCCTTTCGTA
P_{vscN}-*lux*	P1: GCGGAGCTCATCCATTCCGGACCGATGCAT
	P2: GCGGGATCCTGTAAAAAATATGCGCAATGA
flgB^L-RT-qPCR	P1: GTACATCCAGAGGCACTCAAT
	P2: AACTTAGGTCTTTCGCCAAGTA
motY^L-RT-qPCR	P1: TTACGGTTACCAAGCCTTCTC
	P2: TCCTTCCAATACCAGCGTAATC
lafA^L-RT-qPCR	P1: CAACATCATGGGCAACACATC
	P2: GAAGAAGCACCGATTTGGAAAG
flgB-RT-qPCR	P1: CCTGGTTACAAAGCAAGAGGT
	P2: ACGTTGTAGTGGCAGGAATATG
cheZ-RT-qPCR	P1: GATCAACGCATGACCGAAATAG
	P2: CTGCATCCATCGTCTTGTTTG
fliA-RT-qPCR	P1: GACCAGCATGGCAAGTTTAATAG
	P2: CTGAACACTCGGCGGTAAA
q4437_08205-RT-qPCR	P1: CAAATGTAGAACGCGATTACCGTGGG
	P2: GCCGTGATAACCAATCACACCAG
q4437_08215-RT-qPCR	P1: GATTGACCAAGACACGGCAATCACTG
	P2: TAAACGGCTGGTGAGCCTTGAGTG
q4437_08260-RT-qPCR	P1: AGCAAGACTGCCGTTGTATC
	P2: CACACCGAGCTGACGTAAAT
q4437_08275-RT-qPCR	P1: CACCACAGGGTGGGAATTAT
	P2: AAGCCCGAGTTGAGTTTGT
lafI--RT-qPCR	P1: GATCACCCTCTCTTCCGTAATG
	P2: TCTTCCTCTTTGGCGAGTAATG
16s rRNA-RT-qPCR	P1: ACCGCCTGGGGAGTACGGTC
	P2: TTGCGCTCGTTGCGGGACTT
pColdII-*lafI*	P1: CATCATCATCATCATCATATGATGGATTCGACCTACACCATCAT
	P2: GAGGGTACCGAGCTCCATATGCTAATCATTCATTTCCGTTAACATGA
